# Antimicrobial Abietane-Type Diterpenoids from *Plectranthus punctatus*

**DOI:** 10.3390/molecules22111919

**Published:** 2017-11-07

**Authors:** Negera Abdissa, Marcel Frese, Norbert Sewald

**Affiliations:** 1 Department of Chemistry, Organic and Bioorganic Chemistry, Bielefeld University, P.O. Box 100131, Bielefeld 33501, Germany; marcel.frese@uni-bielefeld.de; 2 Department of Chemistry, College of Natural Sciences, Jimma University, P.O. Box 378, Jimma 251, Ethiopia

**Keywords:** antibiotic, benzoquinone, ethnomedicine, diterpenoids, plant-derived natural products

## Abstract

Four new *para*-benzoquinone containing abietane-type diterpenoids (**1**–**4**) along with thirteen known diterpenoids (**5**–**17**) were isolated from the roots of *Plectranthus punctatus*. The structures of the compounds were established by detailed spectroscopic analyses and comparison with literature data. The compounds were tested for their antibacterial and cytotoxic activity and showed significant inhibitory activity against all bacterial strains used, with compounds **6**, **8**, **10**, and **11** showing an inhibition zone for *Staphylococcus warneri* even greater than the reference drug, gentamycin.

## 1. Introduction

*Plectranthus* (family Lamiaceae; subfamily Nepetoideae) is a widespread genus comprising about 300 species worldwide [1]. Although *Plectranthus* species are being recognized for their ornamental value and cultivated for their attractive foliage [2], some are also known for their use in traditional medicinal practice [3] and for their economic value, as they are rich in essential oils, having applications in cosmetics and pharmaceutical products [4]. *Plectranthus* species produce the abietane-type diterpenoids as common secondary metabolites with reported antibacterial [5,6,7], antitumoral [6,7], antifungal [8], insecticidal [9], and antiplasmodial [7] activities.

*Plectranthus punctatus* is a herbaceous perennial plant growing from a tuberous rootstock [10]. It has been reported to be used traditionally as pain killer, anthelmintic, and for medicine after vomiting [10,11,12]. The root of *P*. *punctatus* is being commonly applied by traditional healers in the eastern part of Ethiopia for treatment of stomach pain and wound healings. However, the plant has no previous record of phytochemical analysis, and reports on its biological activity are only limited to ovicidal and larvicidal activity of its crude extract [12].

## 2. Results and Discussion

**Figure 1:**
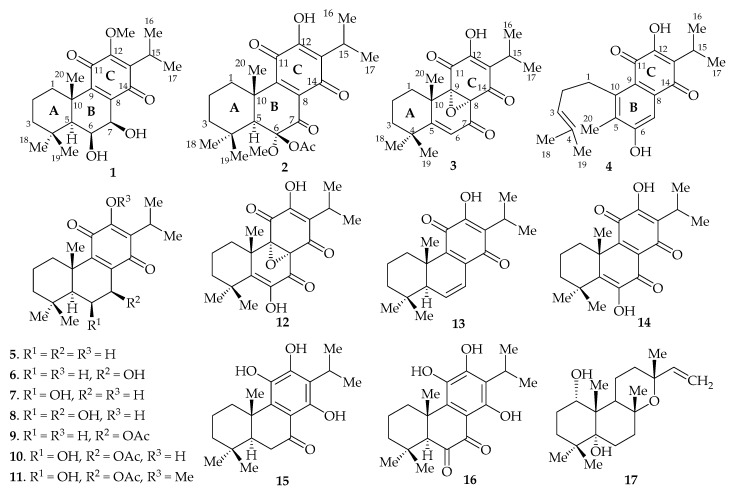
Structures of the isolated compounds.

We report the isolation of four new abietane-type diterpenoids (**1**–**4**), together with thirteen previously known compounds (Figure 1) from the methanol/dichloromethane (1:1) extract of the root of *P*. *punctatus*, and the evaluation of the cytotoxic and antibacterial activities of the compounds. The known diterpenoids were identified as royleanone (**5**) [13], 7β-hydroxyroyleanone (**6**) [14], 6β-hydroxyroyleanone (**7**) [14], 6β,7β-dihydroxyroyleanone (**8**) [15], 7β-acetoxyroyleanone (**9**) [13], 7β-acetoxy-6β-hydroxyroyleanone (**10**) [15], 7β-acetoxy-6β-hydroxy-12-*O*-methylroyleanone (**11**) [15], 8α,9α-epoxycoleon-U-quinone (**12**) [16], 6,7-dehydroroyleanone (**13) [14]**, coleon-U-quinone (**14**) [17], demethylinuroyleanol (**15**) [18], coleon V (**16**) [17], 1α,5α-dihydroxymanoyl oxide (**17**) [19] by comparison with reported spectroscopic data.

Compound **1** was isolated as a yellow amorphous solid. The HR-ESI-MS ion peaks at *m*/*z* 363.2170 for [M + H]^+^ and 385.1981 for [M + Na]^+^ are in agreement with the molecular formula C_21_H_30_O_5_, indicating seven double bond equivalents. The sharp IR absorptions at 1655 and 1640 cm^−1^ are attributed to the presence of conjugated carbonyls and the broad band at 3432 cm^−1^ to hydroxyl groups. The maximum absorption bands at 192 and 270 nm in UV spectrum and ^13^C-NMR signals at δ_C_ 184.9 and 189.3 are in agreement with the presence of a *para*-benzoquinone moiety [15]. The ^1^H-NMR spectrum (Supplementary Material) suggests the presence of an isopropyl substituent at the *para*-benzoquinone as evidenced from the characteristic downfield shifted signal at δ_H_ 3.18 (1H, qq, *J* = 7.1, 7.1 Hz, H-15) and two doublet methyl groups at δ_H_ 1.21 (3H, d, *J* = 7.1 Hz, CH_3_-16), and 1.73 (3H, d, *J* = 7.1 Hz, CH_3_-17), which is consistent with an oxidized C-ring as in abietane-type diterpenoids [15]. The ^1^H-NMR spectrum further shows two oxygenated methine signals at δ_H_ 4.35 (1H, dd, *J* = 3.7, 2.2 Hz) and 4.56 (1H, d, *J* = 2.2 Hz), a tertiary methine signal at δ_H_ 1.54 (1H, d, *J* = 3.7 Hz), three singlet methyl groups (δ_H_ 1.00, 1.27, 1.66), and a set of signals between 1.12 and 2.51, for three mutually coupled (as deduced from COSY experiments) vicinal methylene groups. The presence of one singlet at δ_H_ 3.91, for a methoxy group, was also evident. The ^13^C-NMR spectrum (Supplementary Material) shows 21 carbon signals corresponding to the *para*-benzoquinone moiety (δ_C_ 136.2, 140.5, 150.3, 157.8, 184.9, and 189.3) of ring C, together with two aliphatic quaternary carbons (δ_C_ 34.5, 39.9), four methine groups (δ_C_ 25.3, 49.6, 67.9, 69.9), three methylene groups (δ_C_ 24.4, 39.2, 43.3), five methyl (δ_C_ 19.9, 20.7, 21.0, 22.3, 34.2), and one methoxy (δ_C_ 61.1) carbon atoms. The ^1^H- and ^13^C-NMR spectra of **1** closely resemble those of compound **8**, an abietane-type diterpenoid previously isolated from *P. zeylanicus* [15]. The only notable difference is the absence of a signal for a hydroxyl group which resonates at δ_H_ 7.04 in **8**; instead, a signal for a methoxy group (δ_H_ 3.91; δ_C_ 61.1) is observed in **1**, indicating the presence of a methoxy group at C-12.

**Figure 2:**
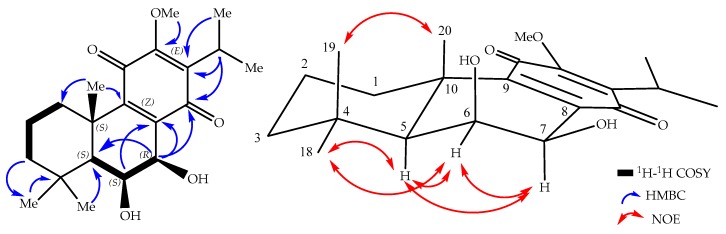
Key ^1^H–^1^H COSY (bold lines), HMBC (blue arrows) and NOE (red arrows) correlations of **1**.

**Figure 3:**
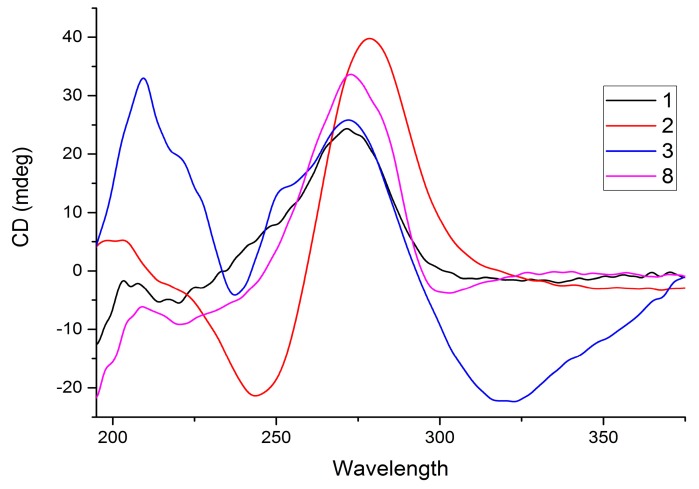
CD spectra for compounds **1**–**3** and **8** (in acetonitrile).

The relative configuration was established based on the NOE experimental data and coupling constants. The NOESY cross peaks from H-6 (δ_H_ 4.35) to H-7 (δ_H_ 4.56), H-5 (δ_H_ 1.45), and H-18 (δ_H_ 1.00) suggest that these hydrogens are cofacial. This is confirmed by the small ^1^H–^1^H coupling constants of H-5 (d, *J*_5,6_ = 3.7 Hz), H-6 (dd, *J*_5,6_ = 3.7 Hz; *J*_6,7_ = 2.2 Hz), and H-7 (d, *J*_6,7_ = 2.2 Hz), indicating *cis*-interactions of the vicinal hydrogens with H-5 and H-7 oriented axially (Figure 2). Therefore, the two methyl groups δ_H_ 1.66 and 1.27, at C-10 and C-4, respectively, were assigned as being oriented axially (β), which is further confirmed by the chemical shift value as a result of 1,3-diaxal interactions, with the hydroxyl group at C-6 [15]. The absolute configuration of abietane-type diterpenoids at C-5 and C-10 is 5*S* and 10*S*, based on biosynthetic consideration [20], which in combination with the already established relative configurations, allowed the absolute configuration of the compound to be assigned as 5*S*,6*S*,7*R*,10*S*. Compound **1** has similar optical activity (${\left[\alpha \right]}_{D}^{20}$ = +24 (*c* 0.5, CH_2_Cl_2_)) and CD Cotton effects (Figure 3) as compound **8** [15]. Based on the above spectroscopic evidence, compound **1** was identified as 6,7-dihydroxy-12-methoxy-11,14-dioxoabieta-8,12-diene, which was given the trivial name 6β,7β-dihydroxy-12-methylroyleanone.

The second compound (**2**) was isolated as a yellow powder. Its HR-ESI-MS data reveal a peak for [M + H]^+^ at *m*/*z* 419.2080 and for [M + Na]^+^ at *m*/*z* 441.1905, both corresponding to a molecular formula of C_23_H_30_O_7_. The UV (λ_max_ 193, 270 nm) and IR (ν_max_ 1736, 1652, 1646 cm^−1^) spectra revealed absorptions for a conjugated carbonyl moiety. The ^1^H-NMR spectrum (Supplementary Material) displays signals for a hydroxyl group at δ_H_ 7.03 (s), an isopropyl group at δ_H_ 3.15 (1H, qq, *J* = 7.3, 7.3 Hz), 1.20 (3H, d, *J* = 7.3 Hz), and 1.19 (3H, d, *J* = 7.3 Hz), an acetyl group at δ_H_ 2.13 (s), a methoxy group at δ_H_ 3.42 (s), a methine at δ_H_ 3.00 (s), three methyl groups at δ_H_ 1.00 (s), 1.36 (s), 1.37 (s), and three mutually coupled (deduced from COSY experiment) methylene groups at δ_H_ 1.27–2.83 (m).

**Figure 4:**
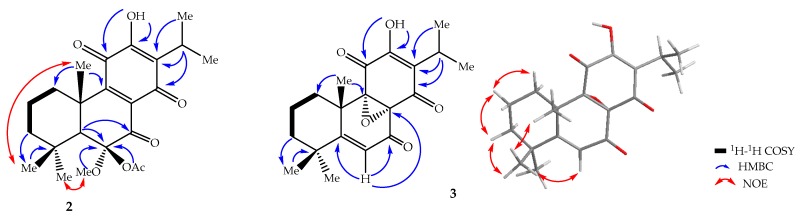
Key ^1^H–^1^H COSY (bold lines); HMBC (blue arrows) and NOE (red arrows) correlations of **2** and **3**.

The ^13^C-NMR spectrum (Supplementary Material) displays signals for 23 carbon atoms assigned to the *para*-benzoquinone moiety (183.8, 183.5, 150.5, 147.6, 138.1, 125.2), a carbonyl group (199.7), three quaternary aliphatic carbons (98.3, 45.2, 32.7), an isopropyl group (24.4, 19.9, 18.8), a methoxy group (52.7), an acetyl residue (20.9, 169.3), three methyl (32.8, 22.0, 18.6), and three methylene (41.6, 36.7, 18.9) groups. These spectroscopic features are similar to those of compound **1**, except for the absence of signals at δ_H_ 4.35 and 4.56 for the two oxygenated methines. Instead, two singlets are observed at δ_H_ 3.42 and 2.13, both integrating for three protons, corresponding to a methoxy and an acetyl group. These are consistent with the presence of extra carbon signals for a methoxy (δ_C_ 52.7) and an acetyl (20.9 and 169.3) group in the ^13^C-NMR spectrum. The upfield chemical shift (δ_H_ 3.42) of the methoxy group is an indication of its attachment to an sp^3^ carbon. The protons of the methoxy and the acetyl groups show HMBC correlations to C-6 (δ_C_ 98.3). A carbonyl signal is present at δ_C_ 199.7 in the ^13^C-NMR spectrum, together with a signal at δ_C_ 98.3, corresponding to a quarternary carbon. This indicates that the two alcohol moieties in **1** resonating at δ_C_ 69.9 (C-6) and 67.9 (C-7) are oxidized to a ketal (δ_C_ 98.3) and a ketone (δ_C_ 199.7), respectively, in compound **2**. The weak HMBC correlation of H-5 (δ_H_ 3.00) with C-6 (δ_C_ 98.3) and strong correlation with C-7 (δ_C_ 199.7) confirm the presence of a ketal and keto moiety at C-6 and C-7, respectively. The relative configuration of **2** was determined to be the same for **1** based on NOESY correlations (Figure 4) observed from 6-OCH_3_ (δ_H_ 3.42) to H-5 (δ_H_ 3.00) and H-18 (δ_H_ 1.00). The latter cross peak also confirms the position of the ketal at C-6. Compound **2** shows a positive specific rotation ((${\left[\alpha \right]}_{D}^{20}$ = +13 (*c* 0.5, CH_2_Cl_2_)) and has a similar CD Cotton effect (Figure 3). Based on the above spectroscopic evidence, the second compound was identified as 6-acetoxy-12-hydroxy-6-methoxy-7,11,14-trioxoabieta-8,12-diene, which was given the trivial name 6β-acetoxy-6α-methoxy-7-oxoroyleanone (**2**).

Compound **3** was obtained as yellow amorphous solid, and its molecular formula C_20_H_24_O_5_ was deduced from HR-ESI-MS (*m*/*z* 345.1745 [M + H]^+^, calcd. for C_20_H_25_O_5_, 345.1702), indicating nine double bond equivalents. The IR spectrum revealed the presence of a hydroxyl group (3379 cm^−1^) and conjugated carbonyls (1729, 1634 cm^−1^). The UV absorptions at 223, 314 nm and the ^13^C-NMR signals at δ_C_ 187.0 and 185.5 support the presence of conjugated carbonyls. The ^1^H-NMR spectrum (Supplementary Material) exhibits an olefinic proton at δ_H_ 6.04 (1H, s, H-6), an isopropyl group at δ_H_ 3.19 (1H, qq, *J* = 7.2, 7.2 Hz, H-15); 1.23 (3H, d, *J* = 7.2 Hz, H-16); 1.21 (3H, d, *J* = 7.2 Hz, H-17), three methyl groups at 1.26 (3H, s, H-18); 1.18 (3H, s, H-19); 1.50 (3H, s, H-20), one hydroxy group at 7.04 (1H, s, 12-OH), and three mutually coupled (according to COSY) methylene groups at δ_H_ 1.44–2.98 (m) (Table 1). The ^13^C-NMR spectrum (Supplementary Material) shows signals corresponding to 20 carbon atoms, including three carbonyl carbons, seven quaternary carbons (three olefinic ones, two oxygenated ones, and two aliphatic ones), two methine groups (one olefinic), three methylene groups and five methyl carbons similar to the abietane-type diterpenoids. However, in this case, it shows signals for conjugated 1,4-dione (187.0, 185.5, 151.2, 128.4) with two oxygen-bearing tertiary carbons at δ_C_ 60.0 and 67.8 that would constitute the *para*-benzoquinone moiety, with the latter two corresponding to epoxy carbon atoms, similar to compound **12** [16]. The HMBC correlations of 12-OH (δ_H_ 7.04) with its neighbors C-11, C-12, C-13, and H-15, with C-12, C-13, C-14, establish the 1,4-dione. The oxygen-bearing tertiary carbons were assigned to C-8 (δ_C_ 60.0) and C-9 (67.8), respectively. This is supported by the HMBC correlation (Figure 4) of H-6, with one of the oxygen bearing carbon, C-8 (60.0), and a carbonyl carbon, C-7 (189.0).

The stereochemistry of **3** was elucidated on the basis of the circular dichroism (CD). The spectrum exhibits a strongly negative Cotton effect at longer wavelength (320 nm) and a strongly positive Cotton effect at shorter wavelength close to 220 and 270 nm, which is similar with the literature precedent for related compounds (**12** and 8α,9α-epoxy-7-oxoroyleanon) having an epoxide ring at a similar position [16,21]. The absolute configurations at the chiral centers are then assigned as 8*S*,9*S*,10*S* in comparison with compound **12** [16,21]. This compound was, therefore, identified as 12-hydroxy-8α,9α-epoxy-7,11,14-trioxoabieta-5,12-diene, and given the trivial name 8α,9α-epoxy-6-deoxycoleon U (**3**).

Compound **4** was isolated as yellow solid with a molecular formula of C_20_H_24_O_4_, as deduced from the HR-ESI-MS ion at *m*/*z* 329.1754 ([M + H]^+^, calcd. for 329.1753), which corresponds to nine double bond equivalents. The UV (λ_max_ 214, 272, 305) and IR (1637, 1570) absorptions indicate the presence of conjugated quinoid moiety [22]. The ^13^C-NMR shows signals for 20 carbon atoms assignable to two carbonyl (181.4, 185.4), eight quaternary (119.9, 125.4, 129.1, 132.7, 135.2, 148.1, 154.2, 159.6), two methylene (28.0, 30.7), three methine (24.6, 123.8, 112.8), and five methyl (11.5, 17.8, 20.0, 20.0, 25.9) groups. The ^1^H-NMR spectrum shows signals typical for an isopropyl group at δ_H_ 3.35 (1H, qq, *J* = 7.1, 7.1 Hz), 1.30 (3H, d, *J* = 7.1 Hz) and 1.30 (3H, d, *J* = 7.1 Hz), together with a singlet corresponding to hydroxy group at δ_H_ 8.05 on the *para*-benzoquinone ring at C-13 and C-12, respectively, as in abietane-type diterpenoids [13]. These substitutions were further confirmed by HMBC correlations of H-15 (δ_H_ 3.35) to C-12, C-13, C-14, C-16, C-17, and 12-OH (δ_H_ 8.05) to C-11, C-12, C-13. It further shows signals for a singlet aromatic proton (δ_H_ 7.69), an olefinic proton (δ_H_ 5.31), two mutually coupled methylene (δ_H_ 2.19, 3.23), one aromatic methyl (δ_H_ 2.31), and two aliphatic methyl (δ_H_ 1.64, 1.75) groups, which is similar to the rearranged abietane-type diterpenoid, 12-hydroxysapriparaquinone [22]. The downfield shifted singlet aromatic proton signal at δ_H_ 7.69 indicates its position *peri* to the carbonyl carbon, and was assigned to H-7, which otherwise substituted with methyl and hydroxyl groups at C-5 (δ_C_ 129.1) and C-6 (δ_C_ 159.6), respectively, confirmed from the HMBC correlation of H-7 with C-5, C-6, C-9, C-14, and H-20 with C-10, C-5, C-6. This substitution is further supported by biosynthetic consideration, that the rearranged diterpenoid is formed by migration of the C-l0 methyl group to C-5 accompanied by fission of ring A [22]. The other substituents with the two mutually coupled methylene protons (from COSY experiment) at δ_H_ 3.23 and 2.19 were assigned to H-1 and H-2, respectively, which are accompanied by an olefinic proton (H-3) showing HMBC correlation to C-1, C-2, C-4, C-18, and C-19. Therefore, the structure of compound **4** was identified as the rearranged abietane-type diterpenoid, 6,12-dihydroxy-sapriparaquinone.

The compounds were evaluated for their antibacterial and cytotoxic activities. The disk diffusion assay was employed to determine the antibacterial activities of the isolated compounds against five bacterial species: *Escherichia coli* (DSMZ1058), *Bacillus subtilis* (DSMZ704), *Micrococcus luteus* (DSMZ1605), *Pseudomonas agarici* (DSMZ11810), and *Staphylococcus warneri* (DSMZ20036) (Table 2). These strains are non-pathogenic correlates of bacteria resistant to most of the first line antibiotics [23,24]. Hence, any substance toxic to these strains can be assumed to be active on the pathogenic relatives, too. The antibacterial activity test (Table 2) indicates that the compounds have antibacterial activities against all the bacterial strains with compounds **1**, **6**, **8**, **10**, **11**, and **14** displaying superior activity. The antibiotic activity (zone of inhibition) of compounds **1**, **6**, **8**, **10**, and **11** is even greater than that of the reference drug (gentamycin) against *S. warneri* (Table 2). Interestingly, compound **9**, which has similar structural features except for the presence of acetyl group at C-7, exhibits less antibiotic activity. This indicates that the presence of aliphatic free hydroxyl groups at C-6 and C-7 may have a positive effect in inhibiting the growth of these bacteria. Compounds **10**, **11**, and **14** also show better antibiotic activity against *M. luteus* in comparison to the reference drug. In general, the activities of these diterpenoids from the traditional medicinal plant *P. punctatus* against both Gram negative and Gram positive bacteria are good, with variable degree of potency between the tested compounds, and provide a scientific basis for the traditional use of the plant.

The human cervix carcinoma cell line KB-3-1 was used for cytotoxicity test with cryptophycin-52 (IC_50_ = 1.3 × 10^−5^ µM) and griseofulvin (IC_50_ = 19 µM) as positive controls, as described in previous reports [25]. Compounds **6**, **7**, **9**, **10**, **11**, and **13** showed marginal cytotoxic activity with IC_50_ values of 50, 49, 13, 42, 52, and 30 µM, respectively, whereas the other compounds showed little or none inhibitory activities.

## 3. Materials and Methods

### 3.1. General Information

Column chromatography was carried out on silica gel (0.06–0.2 mm, Merck, Darmstadt, Germany). Gel filtration was performed on Sephadex LH-20 (GE Healthcare, Uppsala, Sweden). Analytical TLC was performed on Merck pre-coated silica gel 60 F_254_ plates (Merck, Darmstadt, Germany). Preparative HPLC, LaChrom System (Merck Hitachi) equipped with a Phenomenex Jupiter column (10 mm, C18, 300 Å, 250 × 21.1 mm) was used for purification of the compounds. Melting points were measured on B-540 melting point apparatus (Büchi, Flawil, Switzerland). UV spectra were recorded on a UV-3100PC spectrophotometer (VWR International GmbH, Darmstadt, Germany). IR spectra were recorded on a Nicolet 380 FT-IR spectrometer (Thermo Electron Corporation, Madison, WI, USA). High Resolution ESI-MS was done on a Micromass AC-TOFmicro mass spectrometer (Micromass, Agilent Technologies 1200 series, Tokyo, Japan). CD spectra were measured on a JASCO J-810 CD spectrometer (JASCO, Tokyo, Japan). Optical rotations were measured on a P-1020 polarimeter (JASCO, Tokyo, Japan). 1D (^1^H, ^13^C) NMR and 2D (COSY, HSQC, HMBC, NOESY) NMR spectra were recorded on an Avance 500 MHz spectrometer (Bruker, Billerica, MA, USA, at 500 MHz (^1^H) and 125 MHz (^13^C) at 298 K, using the residual solvent peaks (acetone: δ_H_ 2.05, δ_C_ 29.84; CDCl_3_: δ_H_ 7.26, δ_C_ 77.16) as a reference.

### 3.2. Plant Materials

The roots of *P. punctatus* were collected from Gulufa, Amuru District, Horro Guduru Wolega zone, Oromia regional state, Ethiopia in September 2016. The plant material was identified and the voucher specimen (voucher number NA-07) has been deposited in Jimma University Herbarium.

### 3.3. Extraction and Isolation

The air-dried roots (620 g) of *P. punctatus* were milled into powder and then extracted using CH_2_Cl_2_/MeOH (1:1) four times for 24 h at room temperature. The extract was concentrated under vacuum using a rotary evaporator to yield a dark brown residue (37 g, 5.97%). A 35 g portion of the extract was subjected to column chromatography on silica gel (300 g), eluting with petroleum ether containing increasing amounts of ethyl acetate, to afford 36 major fractions of ca. 250 mL each. Fractions 4–10 (5% EtOAc in petroleum ether) were combined and purified by Sephadex LH-20 (eluting with CH_2_Cl_2_/MeOH; 1:1) to give royleanone (**5**, 4.9 mg), 7β-hydroxyroyleanone (**6**, 84.2 mg) and 6,7-dehydroroyleanone (**13**, 7.3 mg); while fractions 11–15 (10% EtOAc in petroleum ether) showed a yellow precipitate that was washed with 100% petroleum ether and further purified on Sephadex LH-20 (eluting with CH_2_Cl_2_/MeOH; 1:1, *v*/*v*) to give 7β-acetoxyroyleanone (**9**, 27.3 mg), 7β-acetoxy-6β-hydroxyroyleanone (**10**, 24.7 mg), and 7β-acetoxy-6β-hydroxy-12-methylroyleanone (**11**, 4.7 mg). Fractions 16–20 (15% ethyl acetate in petroleum ether) showed mixtures of seven compounds, which were combined and subjected to column chromatography (column size: 80 cm length and 4 cm diameter) on silica gel (250 g; eluent: increasing gradient of EtOAc in petroleum ether) followed by Sephadex LH-20 (eluting with CH_2_Cl_2_/MeOH; 1:1, *v*/*v*) yielding compound **1** (4.8 mg), 6β-hydroxyroyleanone (**7**, 5.1 mg) and 1α, 5α-dihydroxymanoyl oxide (**17**, 4.3 mg). The remaining compounds of the same fraction were further purified by RP-HPLC, with a linear gradient solvent system of 20% acetonitrile/water to 100% acetonitrile at a flow rate of 10 mL/min over 60 min, to afford compound **2** (3.8 mg, t_R_ 42 min), 8α,9α-epoxycoleon-U-quinone (**12**, 3.7 mg, t_R_ 36 min), coleon-U-quinone (**14**, 6.2 mg, t_R_ 46 min), and demethylinuroyleanol (**15,** 3.9 mg, t_R_ 39 min). Fractions 25–30 (35% EtOAc in petroleum ether) were combined and chromatographed on Sephadex LH-20 (eluting with CH_2_Cl_2_/MeOH; 1:1, *v*/*v*) followed by further purification using the same RP-HPLC protocol to give compound **3** (4.3 mg, t_R_ 32 min), **4** (6.1 mg, t_R_ 28 min), 6β,7β-dihydroxyroyleanone (**8**, 11.8 mg, t_R_ 38 min), and coleon V (**16**, 4.7 mg, t_R_ 41 min).

*6β,7β-Dihydroxy-12-methylroyleanone* (**1**): Yellow amorphous solid. m.p. 189–191 °C. UV (CH_3_CN): λ_max_ (logε) = 192 (2.65), 270 (2.46) nm. IR (CH_2_Cl_2_) ν_max_ cm^−1^ 3432, 1655, 1640. ${\left[\alpha \right]}_{D}^{20}$ = +24 (*c* 0.5, CH_2_Cl_2_). ^1^H- and ^13^C-NMR (Table 1). ESI-MS (rel. int.): *m*/*z* = 385 (32, [M + Na]^+^), 363 (65, [M + H]^+^). HR-ESI-MS *m*/*z* = 363.2170, [M + H]^+^ (calcd. for C_21_H_30_O_5_, 363.2172).

*6β-Acetoxy-6α-methoxy-7-oxoroyleanone* (**2**): Yellow amorphous solid. m.p. 147–149 °C. UV (CH_3_CN): λ_max_ (log ε) = 193 (2.71), 270 (2.53) nm. IR (CH_2_Cl_2_) ν_max_ cm^−1^ 3342, 1736, 1652, 1646. ${\left[\alpha \right]}_{D}^{20}$ = +13 (*c* 0.5, CH_2_Cl_2_).^1^H- and ^13^C-NMR (Table 1). ESI-MS (rel. int.) *m*/*z* = 441 (13, [M + Na]^+^), 419 (97, [M + H]^+^). HR-ESI-MS *m*/*z* = 419.2080, [M + H]^+^ (calcd. for C_23_H_30_O_7_, 419.2070); 441.1905, [M + Na]^+^.

*8α,9α-Epoxy-6-deoxycoleon U* (**3**): Yellow solid. m.p. 159–161 °C. UV (CH_3_CN): λ_max_ (logε) = 223 (2.63), 314 (2.21) nm. IR (CH_2_Cl_2_) ν_max_ cm^−1^ 3379, 1729, 1634. ${\left[\alpha \right]}_{D}^{20}$ = +8 (*c* 0.5, CH_2_Cl_2_). ^1^H- and ^13^C-NMR (Table 1). ESI-MS (rel.int.): *m*/*z* = 711 (22, [2M + Na]^+^), 367 (37, [M + Na]^+^), 345 (100, [M + H]^+^). HR-ESI-MS *m*/*z* = 345.1745, [M + H]^+^ (calcd. for C_20_H_24_O_5_, 345.1702).

*6,12-Dihydroxysapriparaquinone* (**4**): Yellow solid. m.p. 234–236 °C. UV (CH_3_CN): λ_max_ (logε) = 214 (2.67), 272 (2.62), 30.5 (2.19) nm. IR (CH_2_Cl_2_) ν_max_ cm^−1^ 3332, 1637, 1570. ^1^H- and ^13^C-NMR (Table 1). ESI-MS (rel. int.): *m*/*z* = 351 (21, [M + Na]^+^), 329 (100, [M + H]^+^). HR-ESI-MS *m*/*z* = 329.1754, [M + H]^+^ (calcd. for C_20_H_24_O_4_, 329.1753).

### 3.4. Antimicrobial Assay Using Agar Diffusion Test

The antimicrobial activity of the isolated compounds was investigated in a paper-disk diffusion assay [26] with some modifications: the bacterial strains *Escherichia coli* (DSMZ1058), *Bacillus subtilis* (DSMZ704), *Micrococcus luteus* (DSMZ1605), *Pseudomonas agarici* (DSMZ11810), and *Staphylococcus warneri* (DSMZ20036) were grown on a nutrient agar medium (3 g L^−1^ beef extract, 10 g L^−1^ peptone, and 20 g L^−1^ agar) and the pH was adjusted to 7.2. Twenty milliliters of medium seeded with test organism was poured into sterile Petri dishes (diameter 9 cm). The paper disks (diameter 6 mm) were placed on inoculated agar plates after solidification. Subsequently, the test samples (5 µg/mL each) were applied to the Petri dishes and kept in a refrigerator at 4 °C for 2 h, to allow the loaded substances to diffuse the into the microbial culture. The plates were then incubated for 24 h at 35 °C, and the diameters of the inhibition zones were then measured.

### 3.5. Cytotoxicity Assay

The human cervix carcinoma cell line KB-3-1 was used in the cytotoxicity assay, as described previously [25]. Briefly, the cell line was cultivated as a monolayer in DMEM (Dulbecco’s modified Eagle medium) with glucose (4.5 g/L), l-glutamine, sodium pyruvate, and phenol red, supplemented with 10% fetal bovine serum (FBS) and were maintained at 5.3% CO_2_ and 37 °C in humidified air. The cells (70% confluence) were detached with trypsin-ethylenediaminetetraacetic acid solution (0.05%; 0.02% in DPBS), and placed in sterile 96-well plates in a density of 10,000 cells in 100 μL medium per well. The dilution series of the compounds were prepared from stock solutions in DMSO of concentrations of 100 mM, 50 mM, or 25 mM, and the stock solutions were diluted with culture medium down to pM concentration. The dilution prepared from stock solution was added to the wells and each concentration was tested in at least six replicates. The control contained the same concentration of DMSO as the first dilution. After incubation for 72 h at 37 °C and 5.3% CO_2_-humidified air, 30 μL of an aqueous resazurin solution (175 μM) was added to each well. The cells were incubated at the same conditions for 5 h. Subsequently, the fluorescence was recorded at a wavelength of 588 nm. The IC_50_ values were calculated as a sigmoidal dose response curve using GRAPHPAD PRISM 4.03.

## 4. Conclusions

Four new diterpenoids were isolated together with thirteen known compounds from the dried roots of *P. punctatus*. Some of the compounds show marginal cytotoxic activity against the human cervix carcinoma cell line KB-3-1. However, almost all compounds display interesting antibacterial activity against both Gram positive and Gram negative microorganisms. In particular, six compounds (**1**, **6**, **8**, **10**, **11**, and **14**) show greater zones of inhibition than the reference drug (gentamycin)*.* This could give insight about the potentials of these compounds as lead structures in development of antibacterial drugs.

## Figures and Tables

**Table 1:** ^1^H (500 MHz) and ^13^C (125 MHz) NMR data of compound **1** (in acetone-*d*_6_) and compounds **2**–**4** (in CDCl_3_).

Position	1		2		3		4	
	δ_H_ (m, *J* in Hz)	δ_C_	δ_H_ (m, *J* in Hz)	δ_C_	δ_H_ (m, *J* in Hz)	δ_C_	δ_H_ (m, *J* in Hz)	δ_C_
1	1.18 (m), 2.51 (m)	39.2	1.36 (m), 2.83 (m)	36.7	1.73 (m), 2.98 (m)	33.9	3.23 (m)	30.7
2	1.12 (m), 1.44 (m)	24.4	1.34 (m), 1.64 (m)	18.9	1.23 (m), 1.70 (m)	17.4	2.19 (m)	28.0
3	1.47 (m), 1.50 (m)	43.3	1.42 (m), 1.27 (m)	41.6	1.58 (m), 1.44 (m)	39.1	5.31 (br t, 3.4)	123.8
4		34.5		32.7		37.7		132.7
5	1.54 (d, 3.7)	49.6	3.00 (s)	58.7		169.5		129.1
6	4.35 (dd, 3.7, 2.2)	69.9		98.3	6.04 (s)	120.9		159.6
7	4.56 (d, 2.2)	67.9		199.7		189.0	7.69 (s)	112.8
8		140.5		138.1		60.0		135.2
9		150.3		147.6		67.7		119.9
10		39.9		45.2		41.6		148.1
11		184.9		183.8		187.0		181.4
12		157.8		150.5		151.2		154.2
13		136.2		125.2		128.4		125.4
14		189.3		183.5		185.5		185.4
15	3.18 (qq, 7.1, 7.1)	25.3	3.15 (qq, 7.3, 7.3)	24.4	3.19 (qq, 7.2, 7.2)	24.9	3.35 (qq, 7.1, 7.1)	24.6
16	1.73 (d, 7.1)	20.7	1.19 (d, 7.3)	19.9	1.23 (d, 7.2)	19.2	1.30 (d, 7.1)	20.0
17	1.21 (d, 7.1)	19.9	1.20 (d, 7.3)	19.8	1.21 (d, 7.2)	19.4	1.30 (d, 7.1)	20.0
18	1.00 (s)	34.2	1.00 (s)	32.8	1.26 (s)	30.7	1. 64 (s)	17.8
19	1.27 (s)	22.3	1.37 (s)	22.0	1.18 (s)	32.2	1.75 (s)	25.9
20	1.66 (s)	21.0	1.36 (s)	18.6	1.50 (s)	24.0	2.31 (s)	11.5
12-OMe/OH	3.91 (s)	61.1	7.03 (s)		7.04 (s)		8.05 (s)	
6-OMe			3.42 (s)	52.7				
6-OAc			2.13 (s)	20.9, 169.3				

**Table 2:** Diameter of zone of bacterial growth inhibition (in mm) of the isolated compounds.

Compound	*E. coli* DSMZ1058	*S. warneri* DSMZ20036	*B. subtlis* DSMZ704	*M. luteus* DSMZ1605	*P. agarici* DSMZ11810
**1**	n.a.	22	20	23	21
**2**	n.a.	9	10	14	9
**3**	n.a.	n.a.	7	n.a.	14
**4**	n.a.	n.a.	7	n.a.	8
**6**	8	26	20	18	23
**7**	8	20	19	21	19
**8**	7	28	15	8	20
**9**	7	9	12	9	9
**10**	7	27	25	25	21
**11**	8	26	25	24	21
**13**	7	7	9	9	n.a.
**14**	8	19	13	24	14
**15**	n.a.	18	13	23	16
**16**	8	19	14	23	16
Gentamycin	23	21	26	23	24

n.a.: not active; all values are mean values ± standard deviation of three replicates.

## Supplementary Materials

The mass spectra and NMR data of compounds **1**–**4** are available.
