# Supplementary material for: Antimicrobial Abietane-Type Diterpenoids from Plectranthus punctatus
Source: Molecules. 2017 Nov 7;22(11):1919. doi: 10.3390/molecules22111919 (PMC6150224; doi:10.3390/molecules22111919)
Supplement: Supplementary file 1 [file molecules-22-01919-s001.pdf]

Supporting Information

Antibiotic abietane-type diterpenoids from *Plectranthus punctatus*

Negera Abdissa, Marcel Frese, and Norbert Sewald

Table of contents

Contents **Page**

Spectroscopic data for 6β, 7β-dihydroxy-12-methylroyleanone (1)

Figure S1: ^{1}H NMR spectrum of 6β, 7β-dihydroxy-12-methylroyleanone (**1**) S2
Figure S2: ^{13}C NMR spectrum of 6β, 7β-dihydroxy-12-methylroyleanone (**1**) S2
Figure S3: ^{13}C DEPT NMR spectrum of 6β, 7β-dihydroxy-12-methylroyleanone (**1**) S3
Figure S4: COSY spectrum of 6β, 7β-dihydroxy-12-methylroyleanone (**1**) S3
Figure S5: HMQC spectrum of 6β, 7β-dihydroxy-12-methylroyleanone (**1**) S4
Figure S6: HMBC spectrum of 6β, 7β-dihydroxy-12-methylroyleanone (**1**) S4
Figure S7: NOESY spectrum of 6β, 7β-dihydroxy-12-methylroyleanone (**1**) S5
Figure S8: HRESIMS spectra of 6β, 7β-dihydroxy-12-methylroyleanone (**1**) S5

Spectroscopic data for 6β-acetoxy-6α-methoxy-7-oxoroyleanone (2)

Figure S9: ^{1}H NMR spectrum of 6β-acetoxy-6α-methoxy-7-oxoroyleanone (**2**) S6
Figure S10: ^{13}C NMR spectrum of 6β-acetoxy-6α-methoxy-7-oxoroyleanone (**2**) S6
Figure S11: COSY spectrum of 6β-acetoxy-6α-methoxy-7-oxoroyleanone (**2**) S7
Figure S12: HMQC spectrum of 6β-acetoxy-6α-methoxy-7-oxoroyleanone (**2**) S7
Figure S13: HMBC spectrum of 6β-acetoxy-6α-methoxy-7-oxoroyleanone (**2**) S8
Figure S14: HRESIMS spectra of 6β-acetoxy-6α-methoxy-7-oxoroyleanone (**2**) S8

Spectroscopic data for 8α,9α-epoxy-6-deoxycoleon U (3)

Figure S15: ^{1}H NMR spectrum of 8α,9α-epoxy-6-deoxycoleon U (**3**) S9
Figure S16: ^{13}C NMR spectrum of 8α,9α-epoxy-6-deoxycoleon U (**3**) S9
Figure S17: COSY spectrum of 8α,9α-epoxy-6-deoxycoleon U (**3**) S10
Figure S18: NOESY spectrum of 8α,9α-epoxy-6-deoxycoleon U (**3**) S10
Figure S19: HMQC spectrum of 8α,9α-epoxy-6-deoxycoleon U (**3**) S11
Figure S20: HMBC spectrum of 8α,9α-epoxy-6-deoxycoleon U (**3**) S11
Figure S21: HRESIMS spectra of 8α,9α-epoxy-6-deoxycoleon U (**3**) S12
Figure S22: IR spectra of 8α,9α-epoxy-6-deoxycoleon U (**3**) S12

Spectroscopic data for 6,12-dihydroxysapriparaquinone (4)

Figure S23: ^{1}H NMR spectrum of 6,12-dihydroxysapriparaquinone (**4**) S13
Figure S24: ^{13}C NMR spectrum of 6,12-dihydroxysapriparaquinone (**4**) S13
Figure S25: ^{13}C DEPT NMR spectrum of 6,12-dihydroxysapriparaquinone (**4**) S14
Figure S26: COSY spectrum of 6,12-dihydroxysapriparaquinone (**4**) S14
Figure S27: HMQC spectrum of 6,12-dihydroxysapriparaquinone (**4**) S15
Figure S28: HMBC spectrum of 6,12-dihydroxysapriparaquinone (**4**) S15
Figure S29: HRESIMS spectra 6,12-dihydroxysapriparaquinone (**4**) S16

Spectroscopic data for 6β, 7β-dihydroxy-12-methylroyleanone (1)

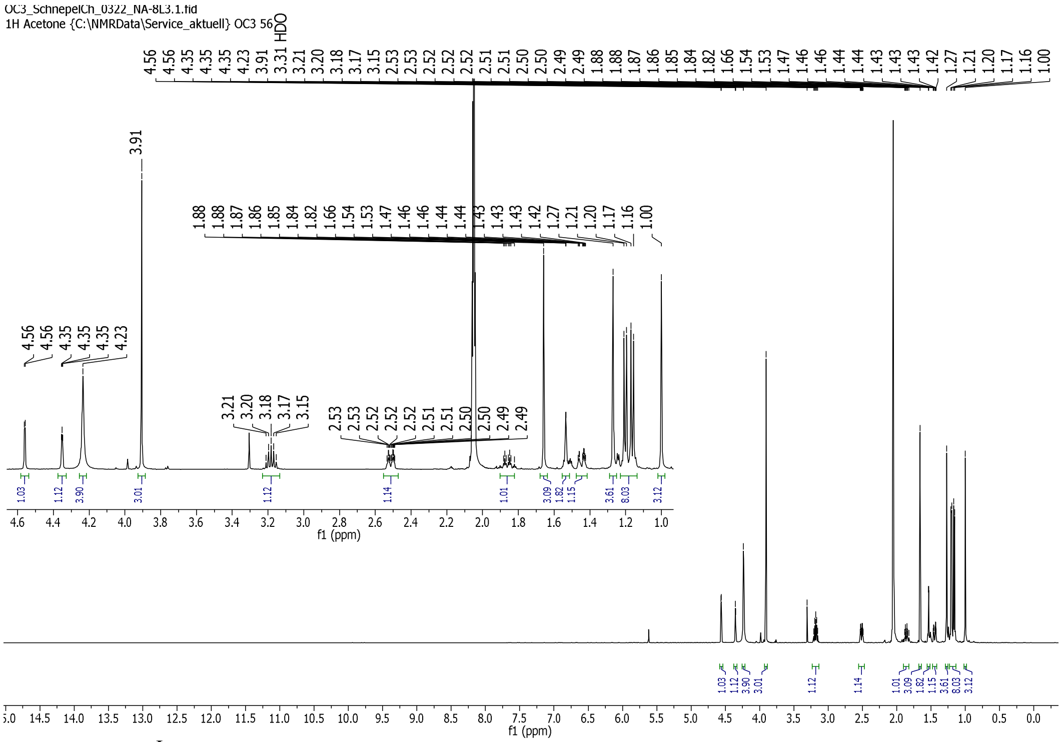

Figure S1. The ^{1}H NMR spectrum of 6β, 7β-dihydroxy-12-methylroyleanone (**1**) observed at 500 MHz in acetone-d_6 at 25 °C. Assignments are given in Table 1.

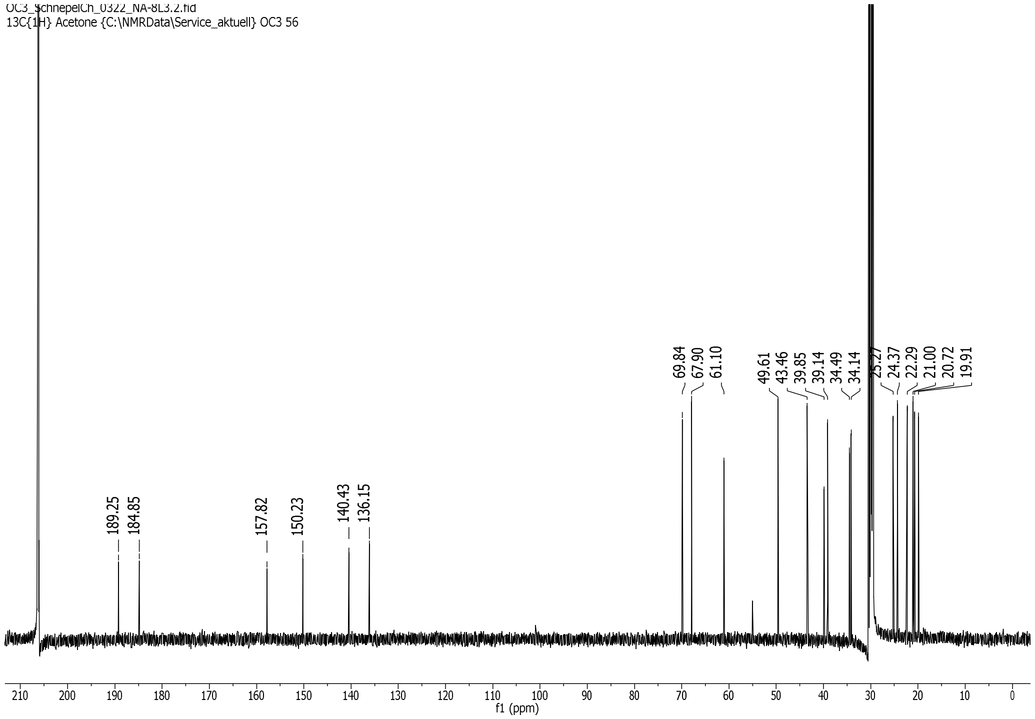

Figure S2. The ^{13}C NMR spectrum of 6β, 7β-dihydroxy-12-methylroyleanone (**1**) observed at 125 MHz in acetone-d_6 solution at 25 °C. Assignments are given in Table 1.

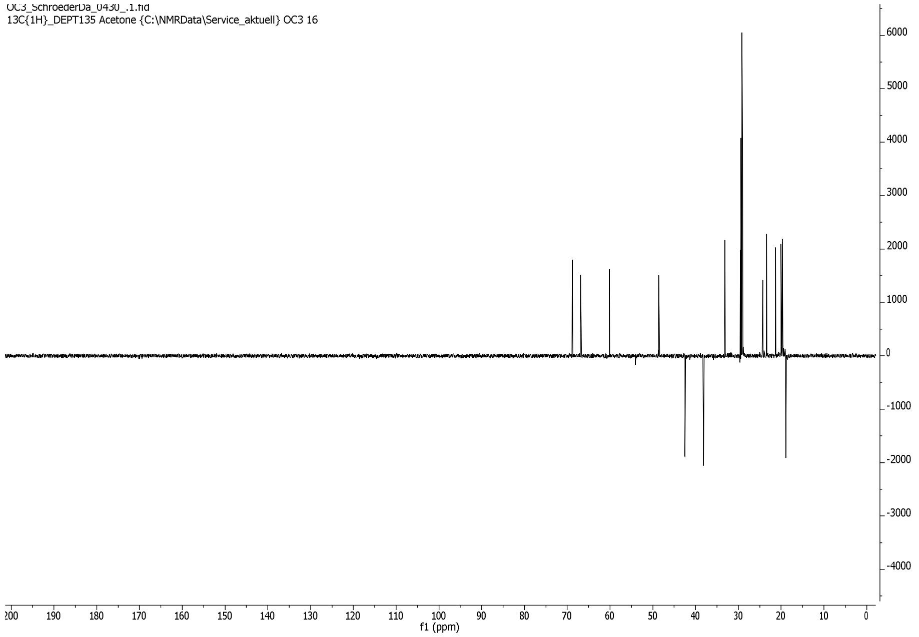

Figure S3. The ^{13}C DEPT NMR spectrum of 6β, 7β-dihydroxy-12-methylroyleanone (**1**) observed at 125 MHz in acetone-d_6 solution at 25 °C.

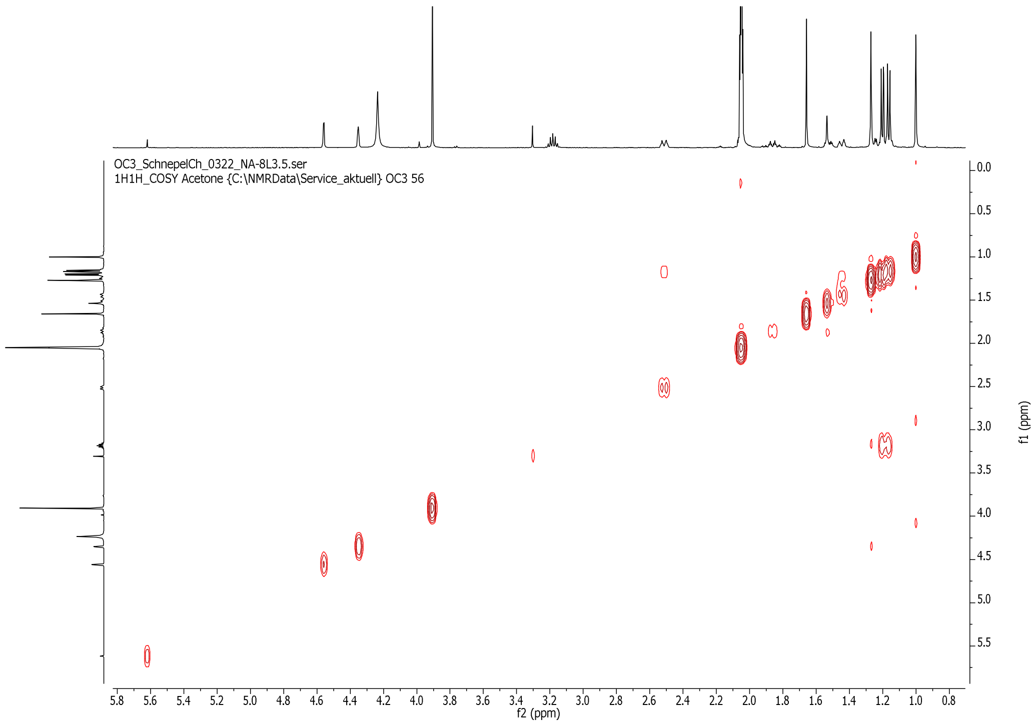

Figure S4. The COSY spectrum of 6β, 7β-dihydroxy-12-methylroyleanone (**1**) observed at 500 MHz in acetone-d_6 solution at 25 °C.

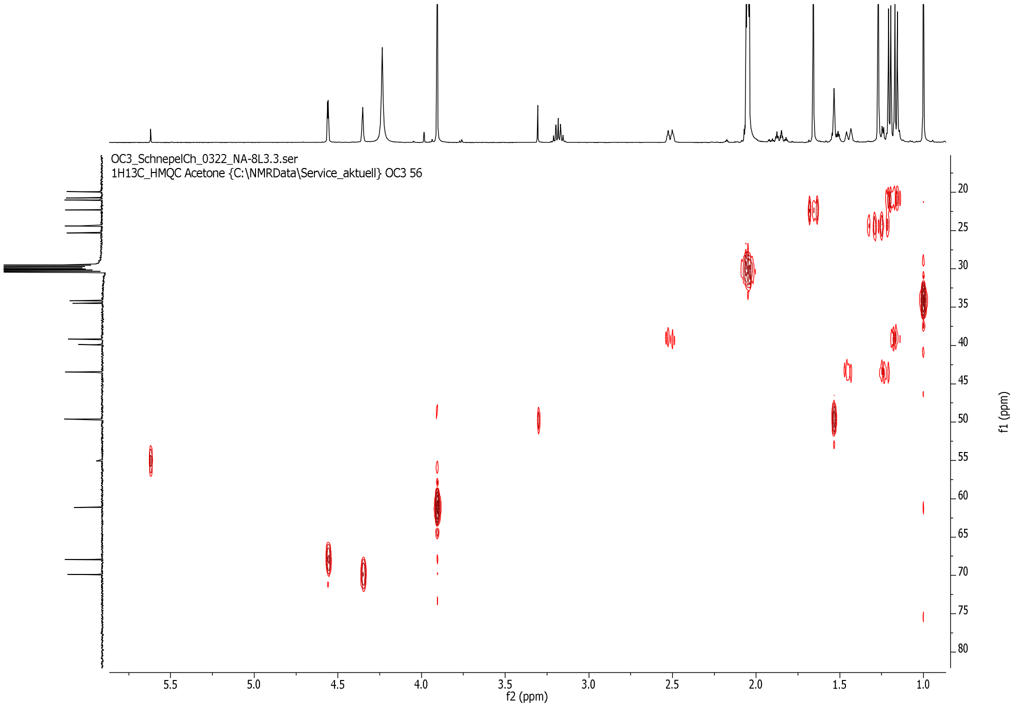

Figure S5. The HSQC spectrum of 6β, 7β-dihydroxy-12-methylroyleanone (**1**) observed at 500 and 125 MHz in acetone-d_6 solution at 25 °C.

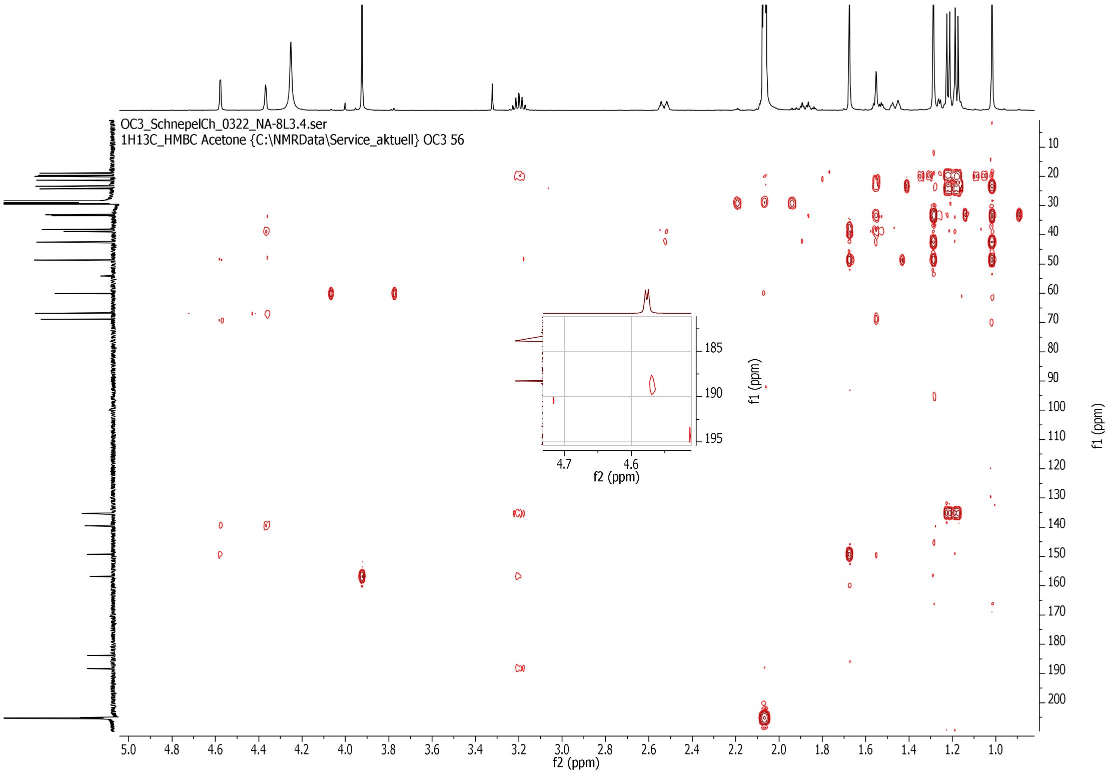

Figure S6. The HMBC spectrum of 6β, 7β-dihydroxy-12-methylroyleanone (**1**) observed at 500 and 125 MHz in acetone-d_6 solution at 25 °C.

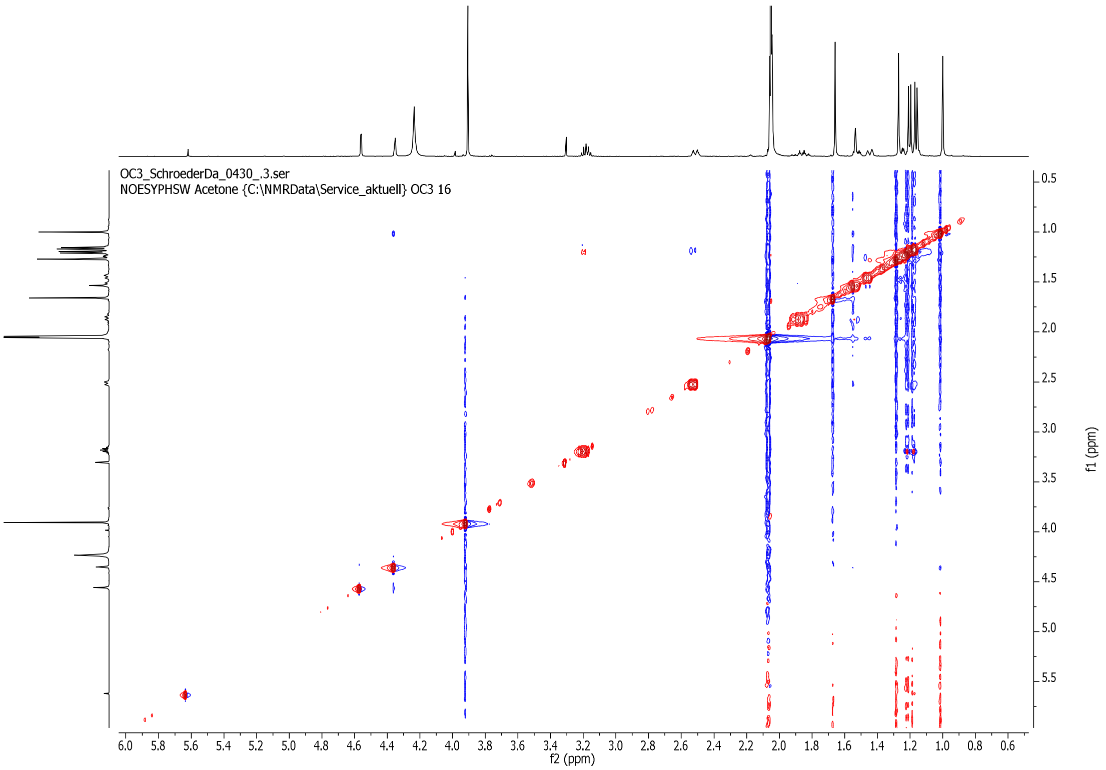

Figure S7. The NOESY spectrum of 6β, 7β-dihydroxy-12-methylroyleanone (**1**) observed at 500 MHz in acetone-d_6 solution at 25 °C.

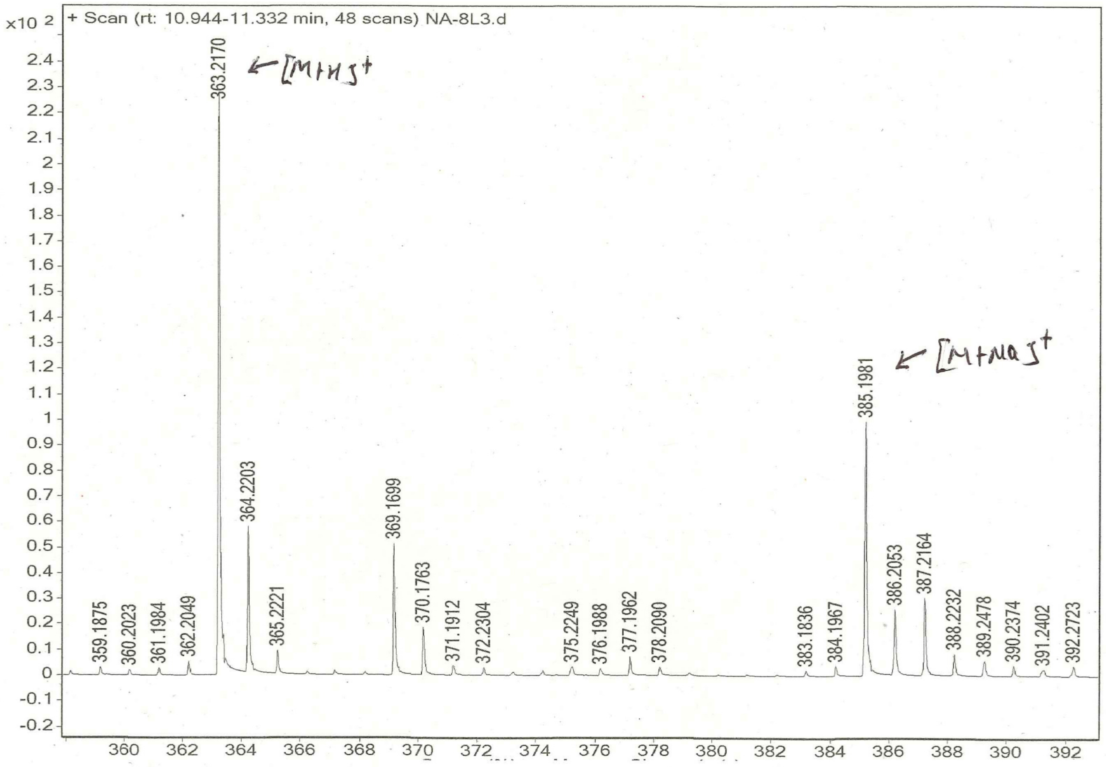

Figure S8. The HRESIMS of 6β, 7β-dihydroxy-12-methylroyleanone (**1**).

Spectroscopic data for 6β-acetoxy-6α-methoxy-7-oxoroyleanone (2)

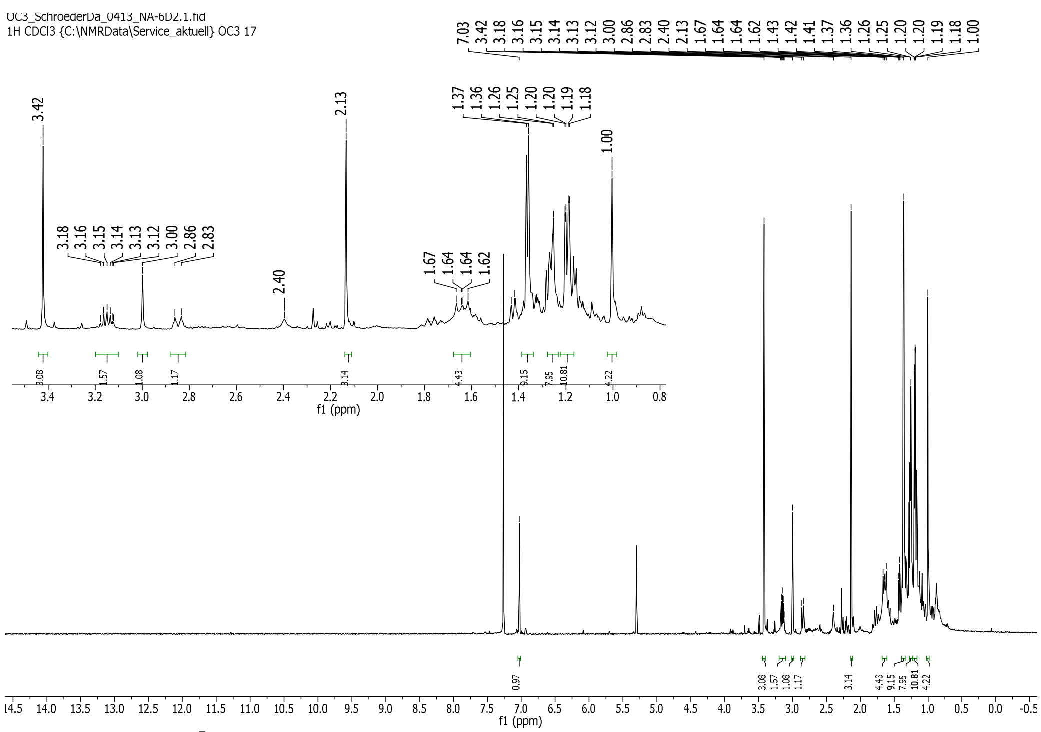

Figure S9. The ^{1}H NMR of 6β-acetoxy-6α-methoxy-7-oxoroyleanone (**2**) observed at 500 MHz in $CDCl_3$ solution at 25 °C. Assignments are given in Table 1.

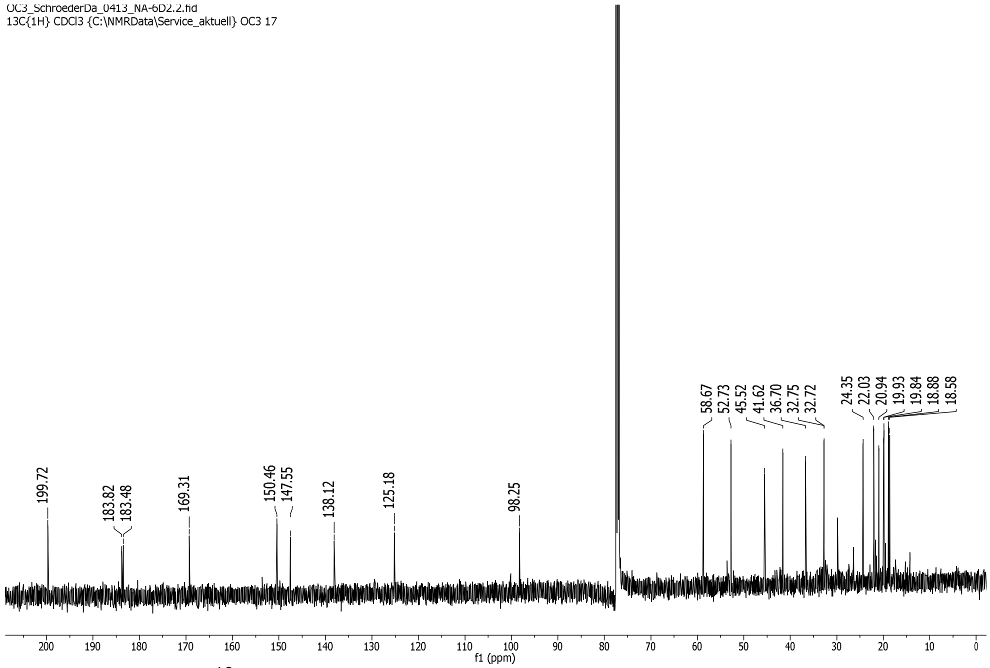

Figure S10. The ^{13}C NMR spectrum of 6β-acetoxy-6α-methoxy-7-oxoroyleanone (**2**) observed at 125 MHz in $CDCl_3$solution at 25 °C. Assignments are given in Table 1.

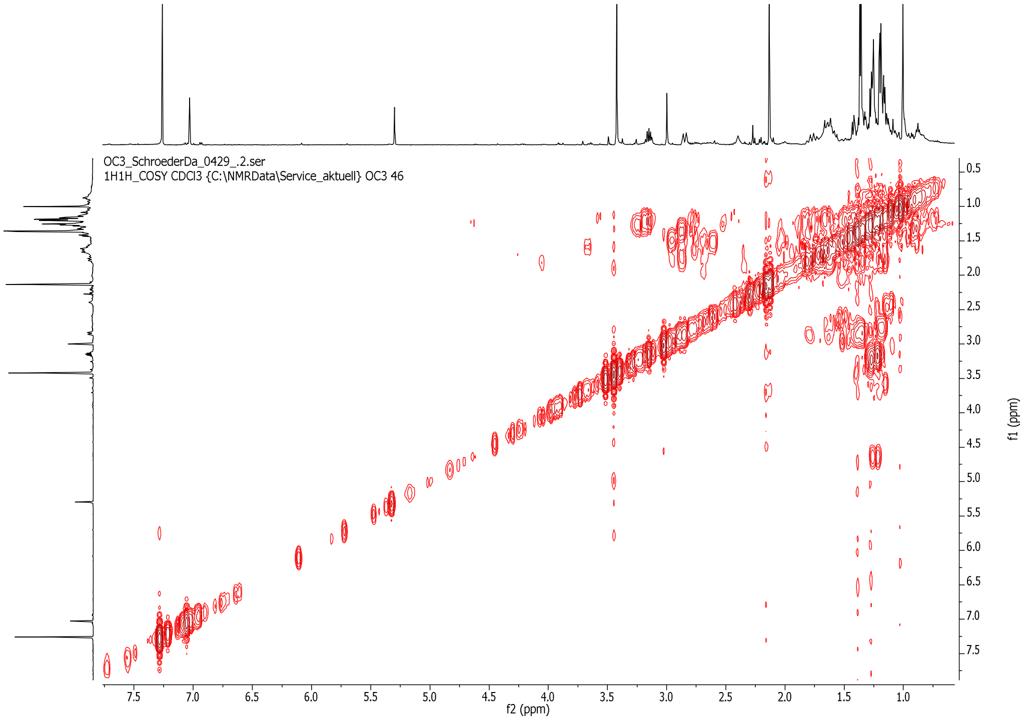

Figure S11. The COSY spectrum of 6β-acetoxy-6α-methoxy-7-oxoroyleanone (**2**) observed at 500 MHz in $CDCl_3$ solution at 25 °C.

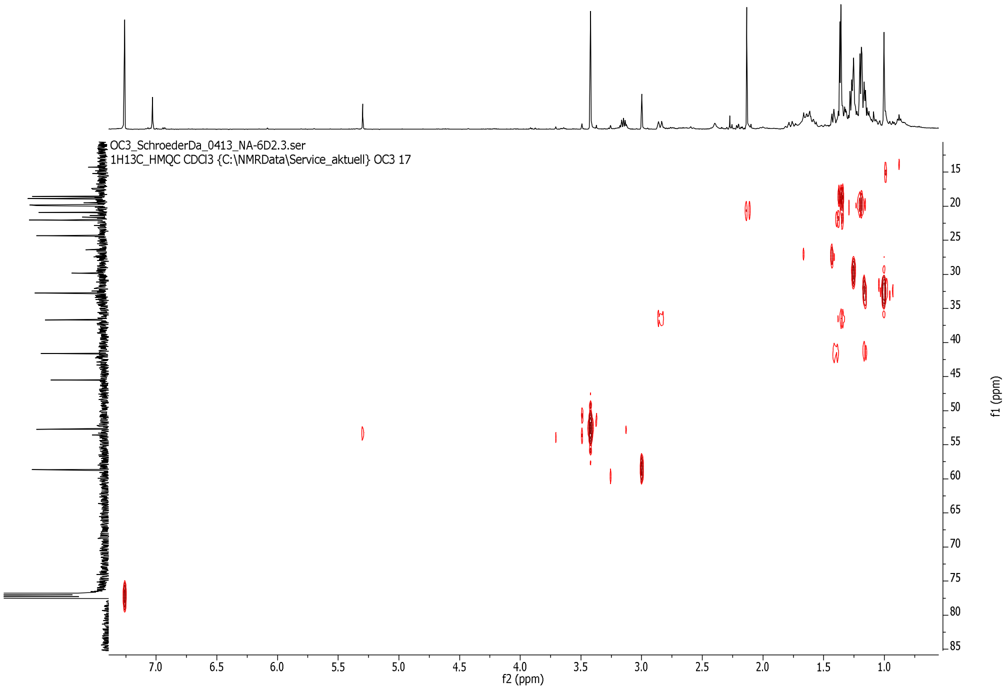

Figure S12. The HSQC spectrum of 6β-acetoxy-6α-methoxy-7-oxoroyleanone (**2**) observed at 500 and 125 MHz in $CDCl_3$ solution at 25 °C.

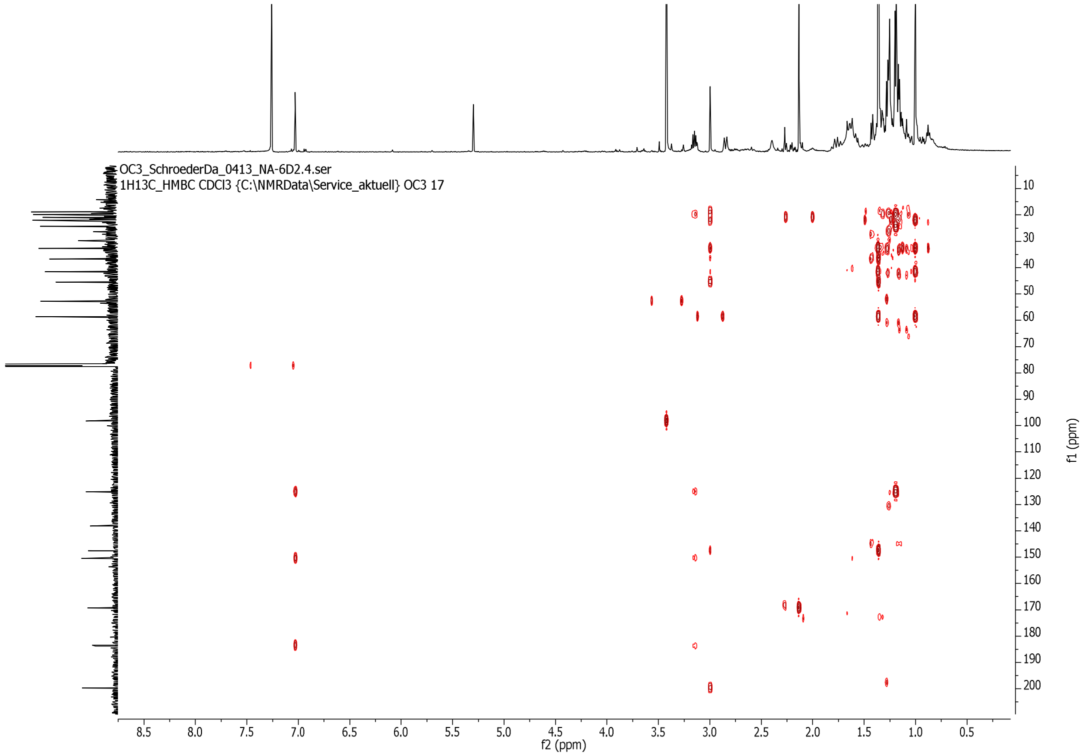

Figure S13. The HMBC spectrum of 6β-acetoxy-6α-methoxy-7-oxoroyleanone (**2**) observed at 500 and 125 MHz in $CDCl_3$ solution at 25 °C.

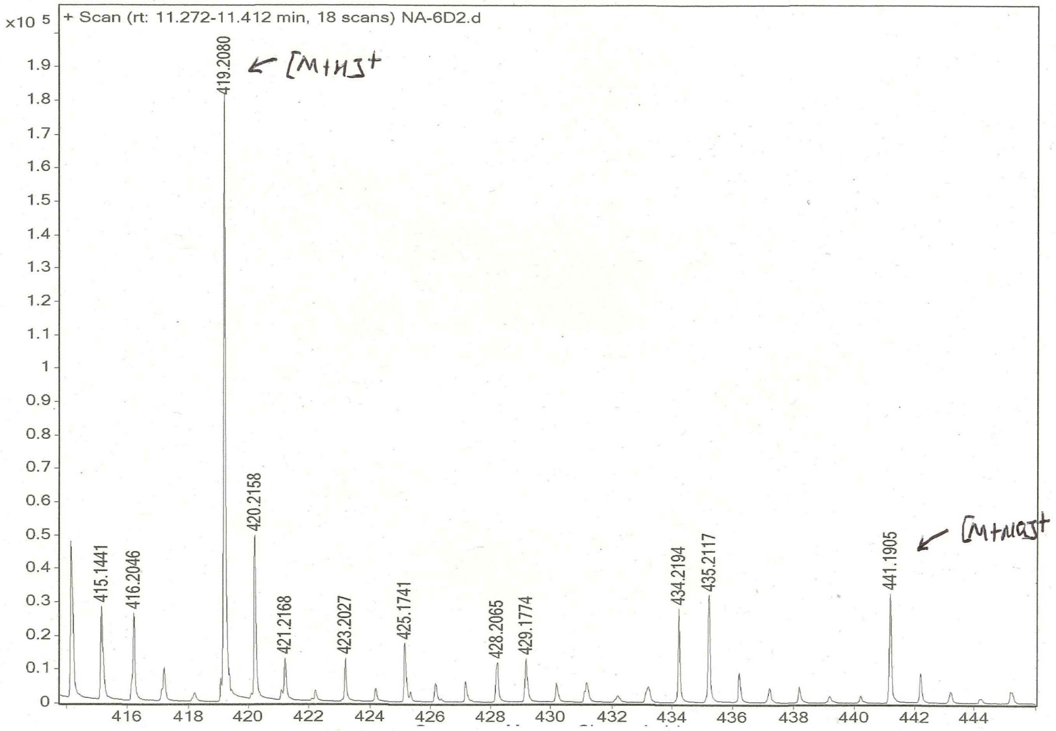

Figure S14. The HRESIMS of 6β-acetoxy-6α-methoxy-7-oxoroyleanone (**2**)

Spectroscopic data for 8α,9α-epoxy-6-deoxycoleon U (3)

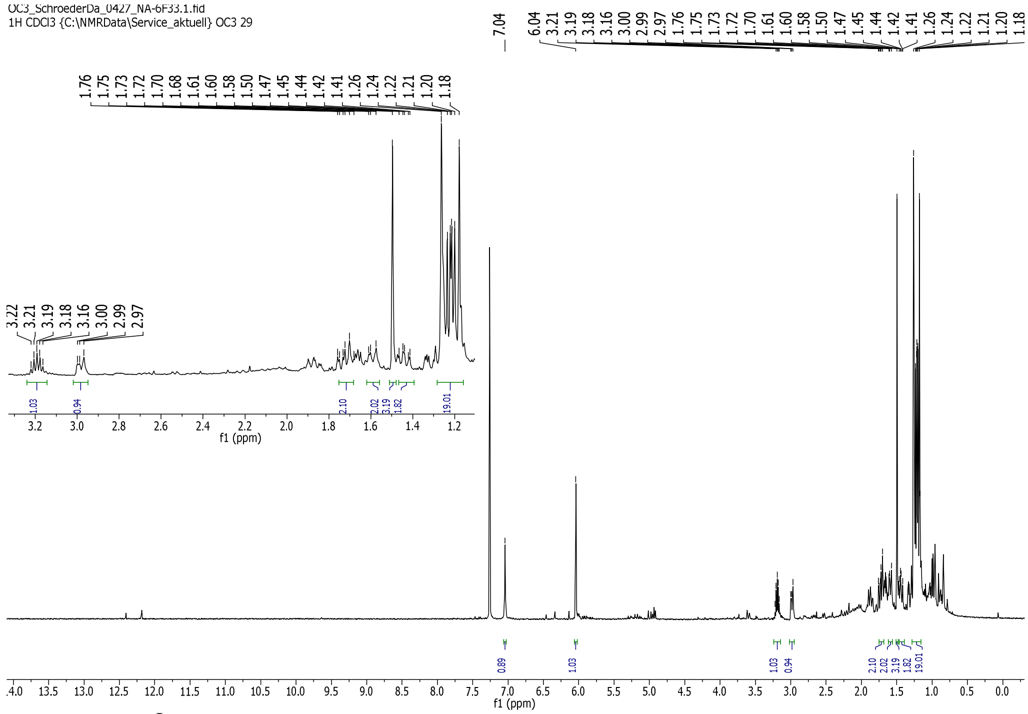

Figure S15. The ^{1}H NMR of 8α,9α-epoxy-6-deoxycoleon U (**3**) observed at 500 MHz in $CDCl_3$ solution at 25 °C. Assignment is given in Table 1.

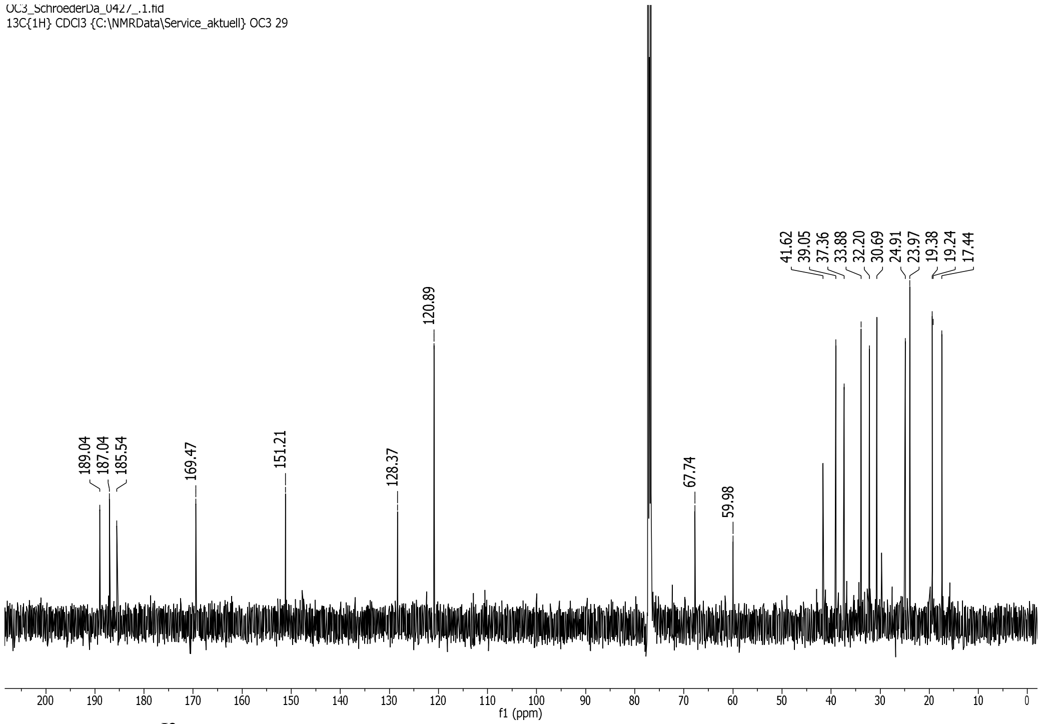

Figure S16. The ^{13}C NMR spectrum of 8α,9α-epoxy-6-deoxycoleon U (**3**) observed at 125 MHz in $CDCl_3$ solution at 25 °C. Assignment is given in Table 1.

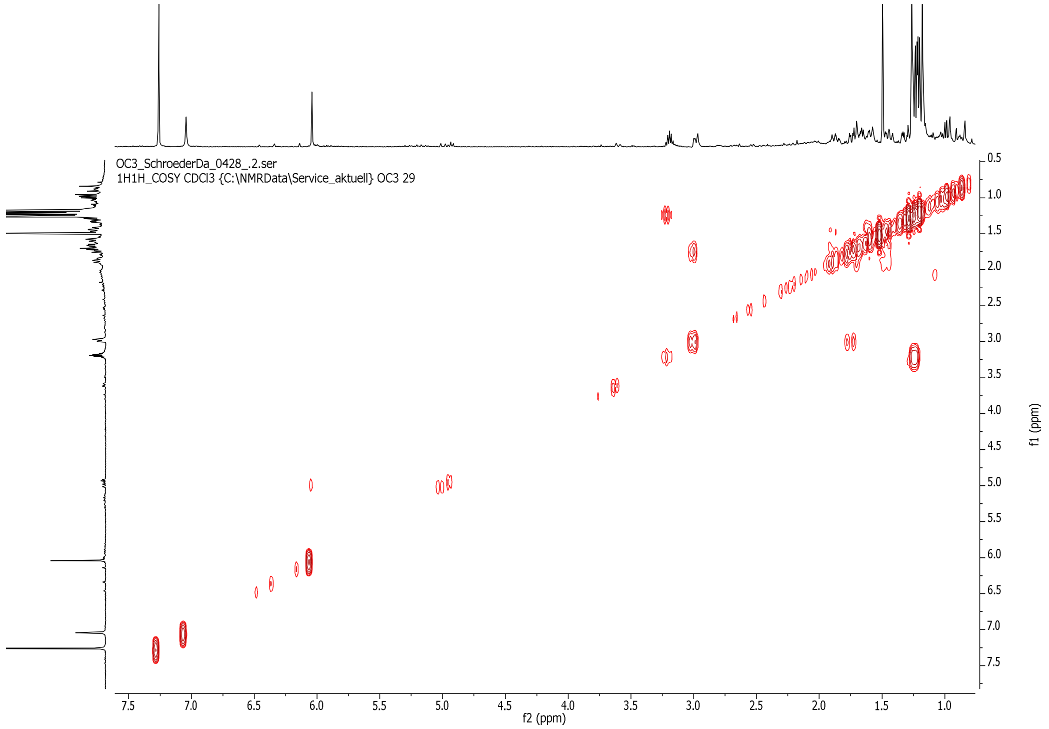

Figure S17. The COSY spectrum of 8α,9α-epoxy-6-deoxycoleon U (**3**) observed at 500 MHz in $CDCl_3$ solution at 25 °C.

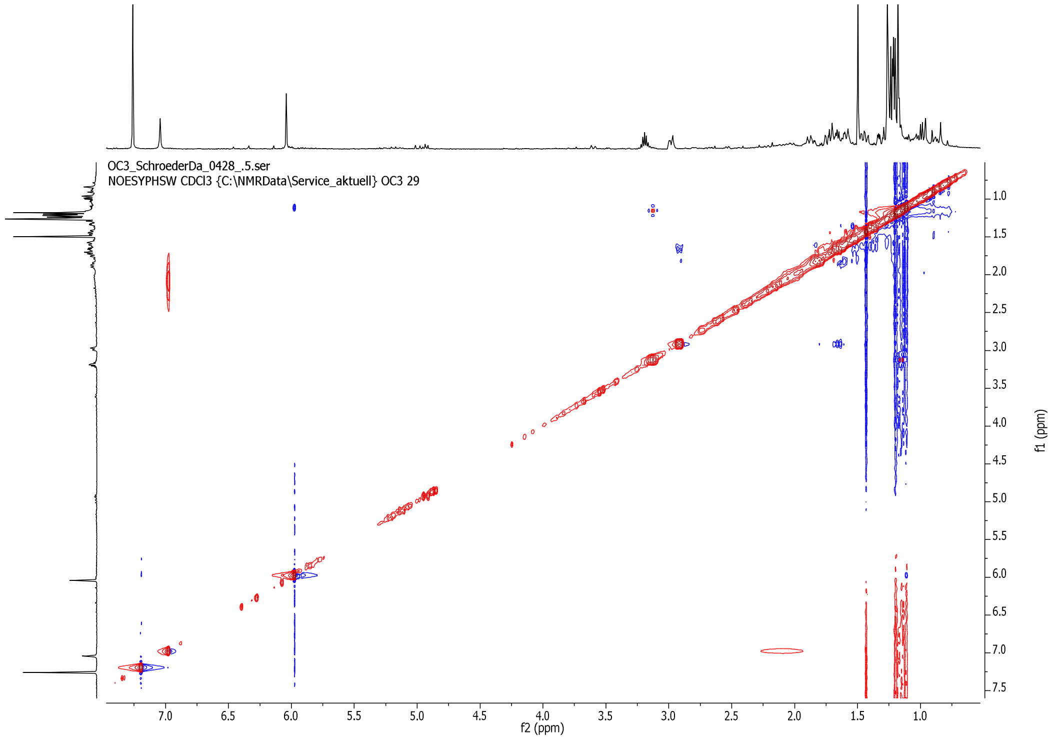

Figure S18. The NOESY spectrum of 8α,9α-epoxy-6-deoxycoleon U (**3**) observed at 500 MHz in $CDCl_3$ solution at 25 °C.

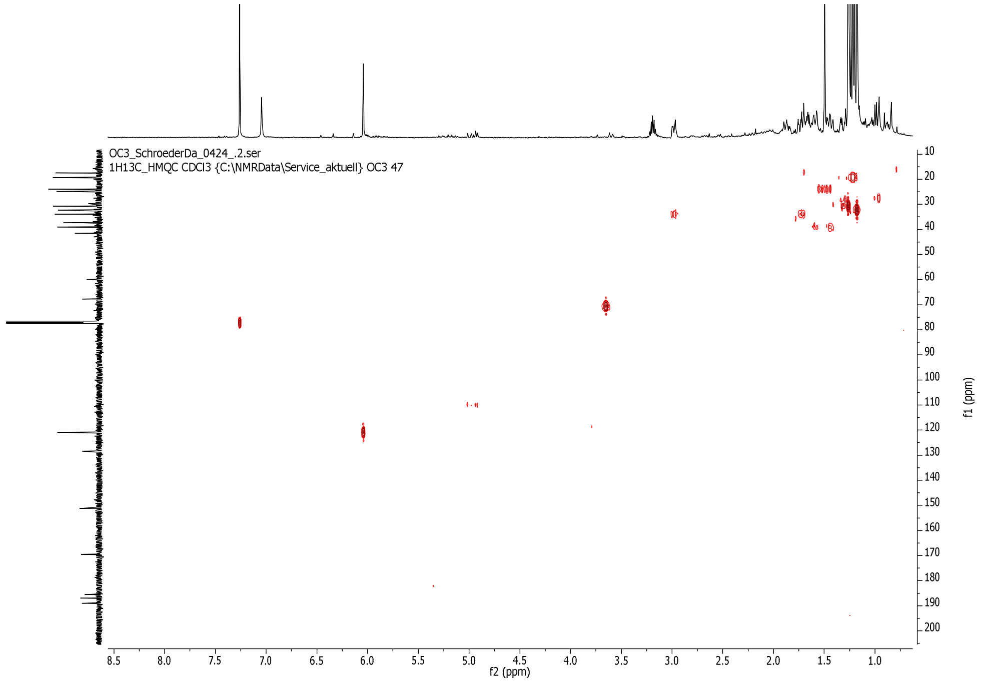

Figure S19. The HSQC spectrum of 8α,9α-epoxy-6-deoxycoleon U (**3**) observed at 500 and 125 MHz in $CDCl_3$ solution at 25 °C.

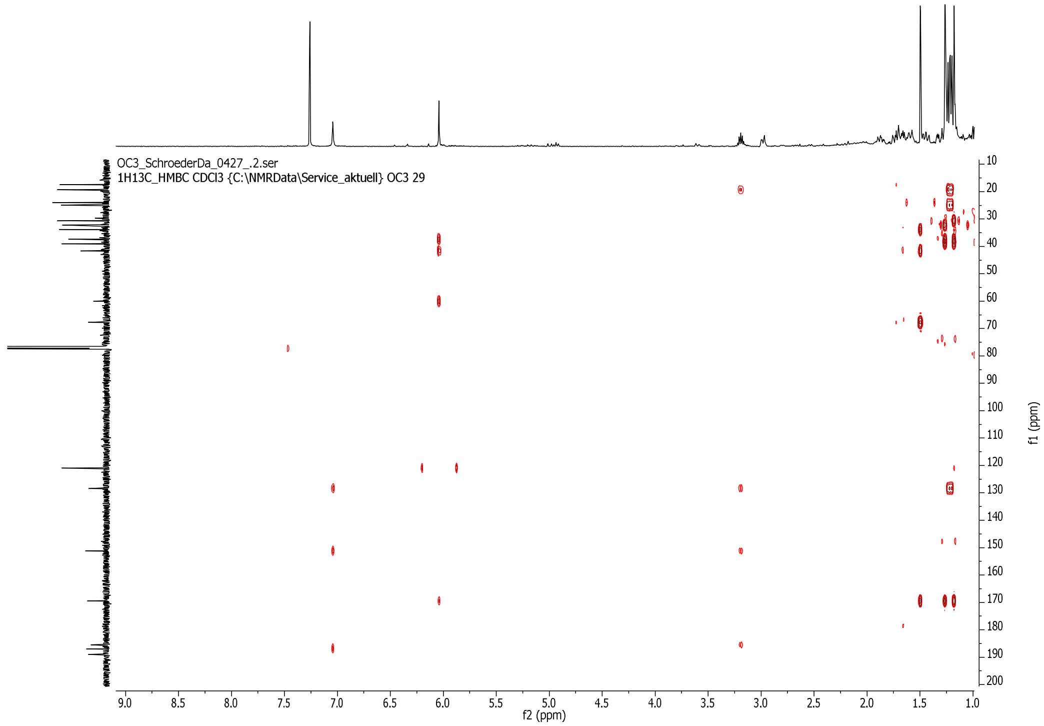

Figure S20. The HMBC spectrum of 8α,9α-epoxy-6-deoxycoleon U (**3**) observed at 500 and 125 MHz in $CDCl_3$ solution at 25 °C.

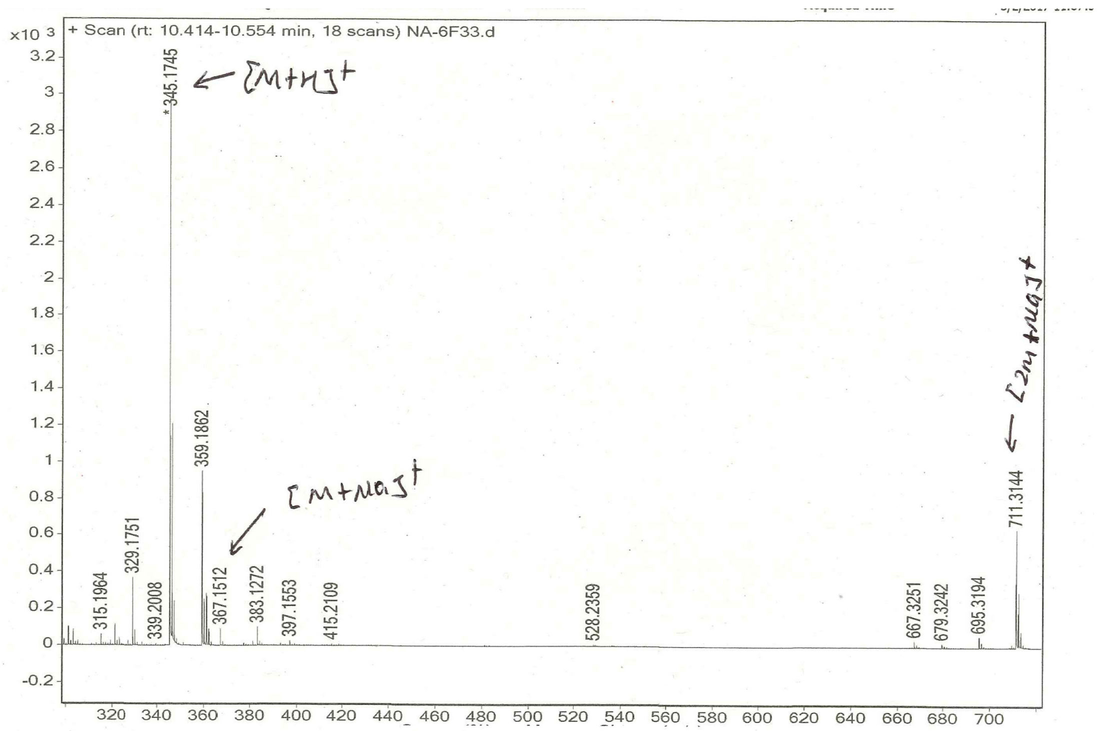

Figure S21. The HRESIMS of 8α,9α-epoxy-6-deoxycoleon U (**3**).

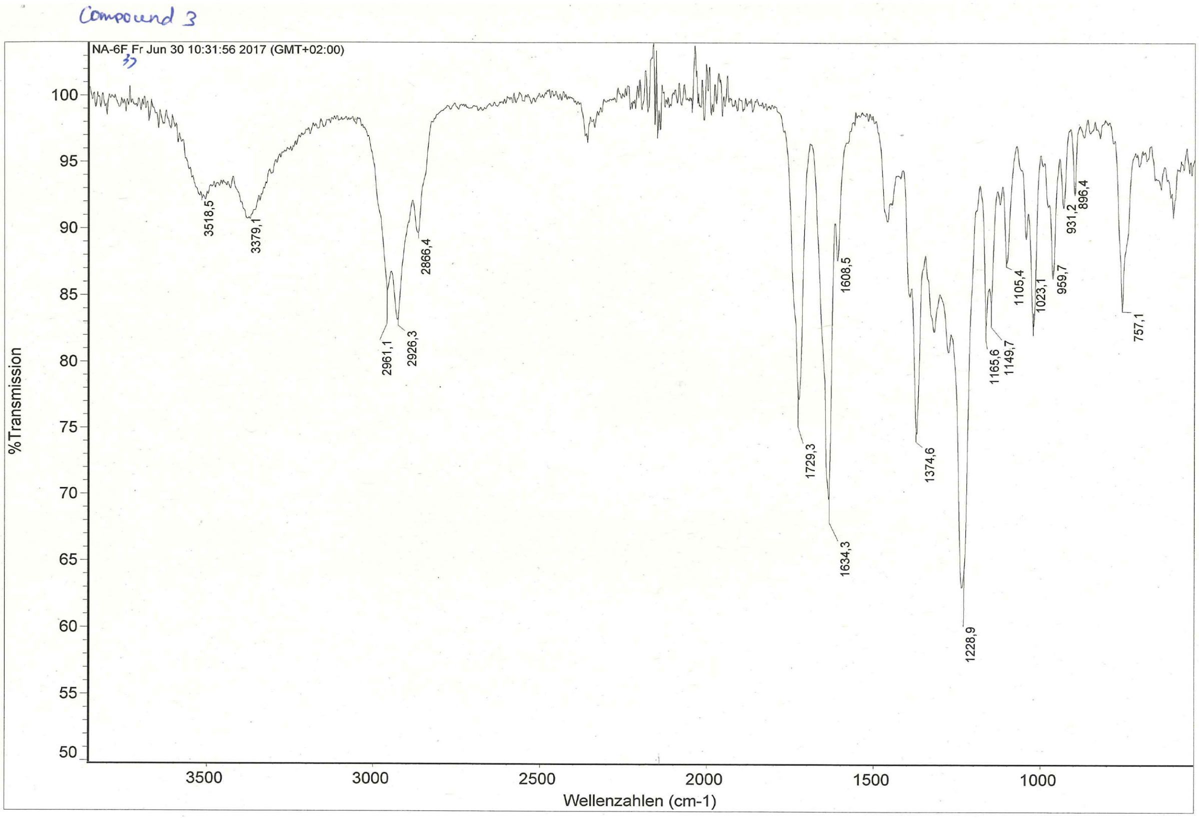

Figure S22. The IR spectra of 8α,9α-epoxy-6-deoxycoleon U (**3**).

Spectroscopic data for 6,12-dihydroxysapriparaquinone (4)

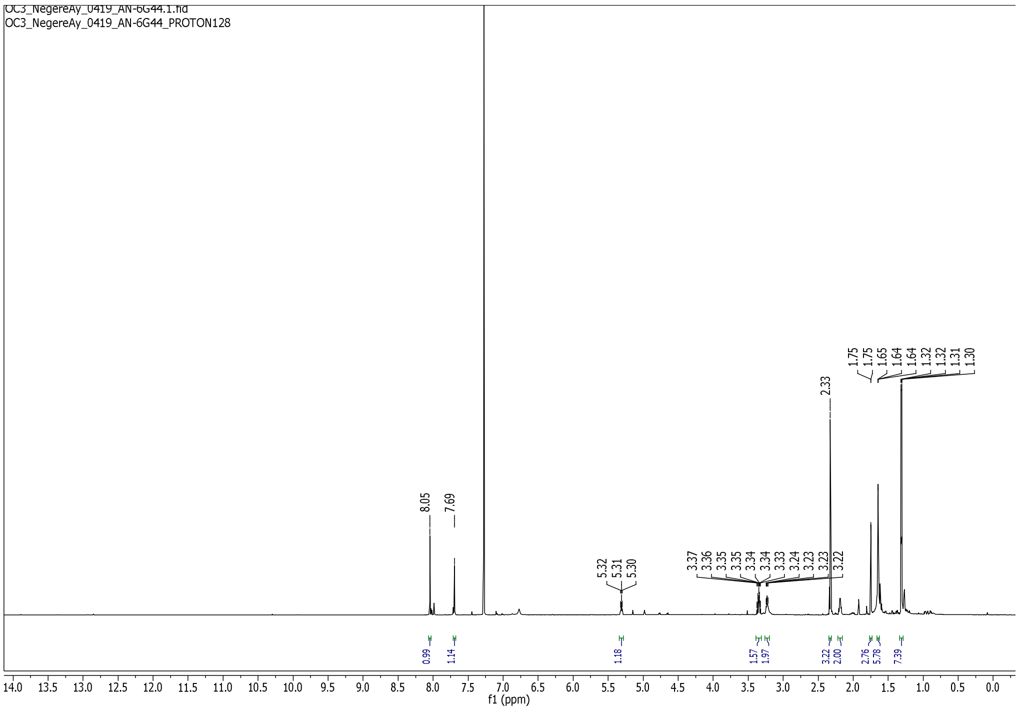

Figure S23. The [I]H NMR of 6,12-dihydroxysapriparaquinone (**4**) observed at 500 MHz in $CDCl_3$ solution at 25 °C. Assignment is given in Table 1.

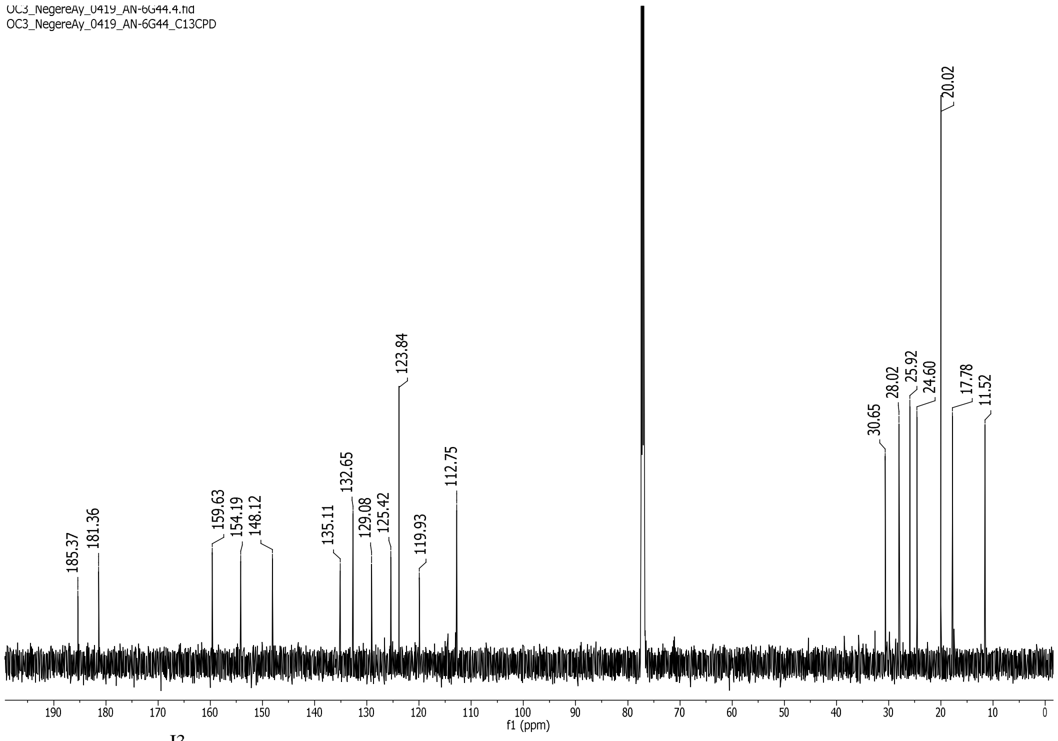

Figure S24. The [I3]C NMR spectrum 6,12-dihydroxysapriparaquinone (**4**) observed at 125 MHz in $CDCl_3$ solution at 25 °C. Assignment is given in Table 1.

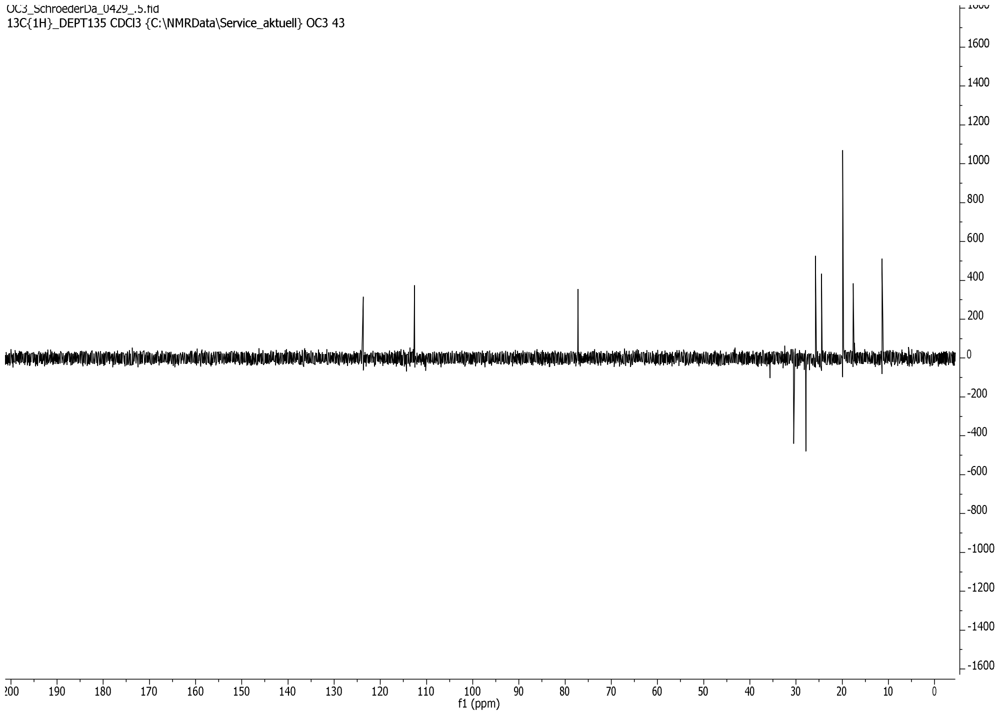

Figure S25. The ^{13}C DEPT NMR spectrum 6,12-dihydroxysapriparaquinone (**4**) observed at 125 MHz in $CDCl_3$ solution at 25 °C.

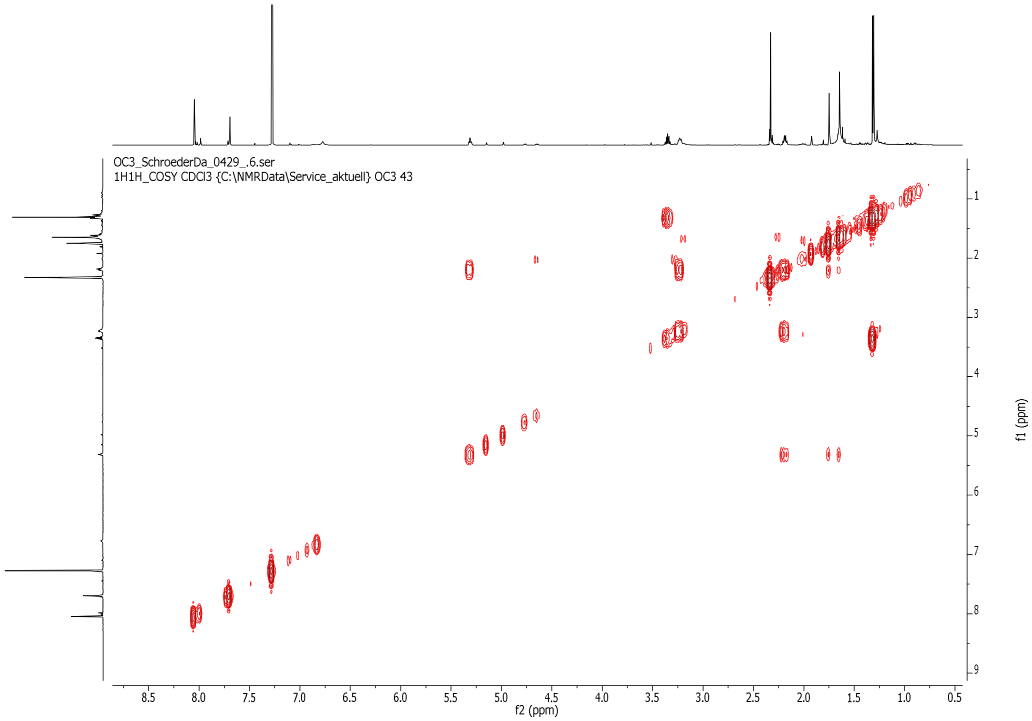

Figure S26. The COSY spectrum of 6,12-dihydroxysapriparaquinone (**4**) observed at 500 MHz in $CDCl_3$ solution at 25 °C.

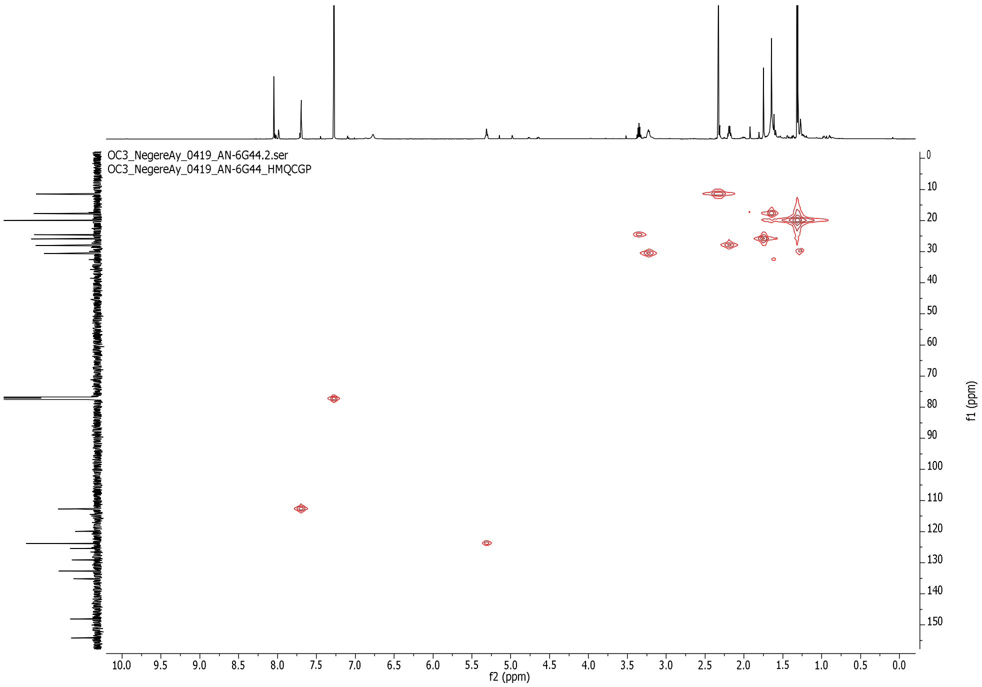

Figure S27. The HSQC spectrum of 6,12-dihydroxysapriparaquinone (**4**) observed at 500 and 125 MHz in $CDCl_3$ solution at 25 °C.

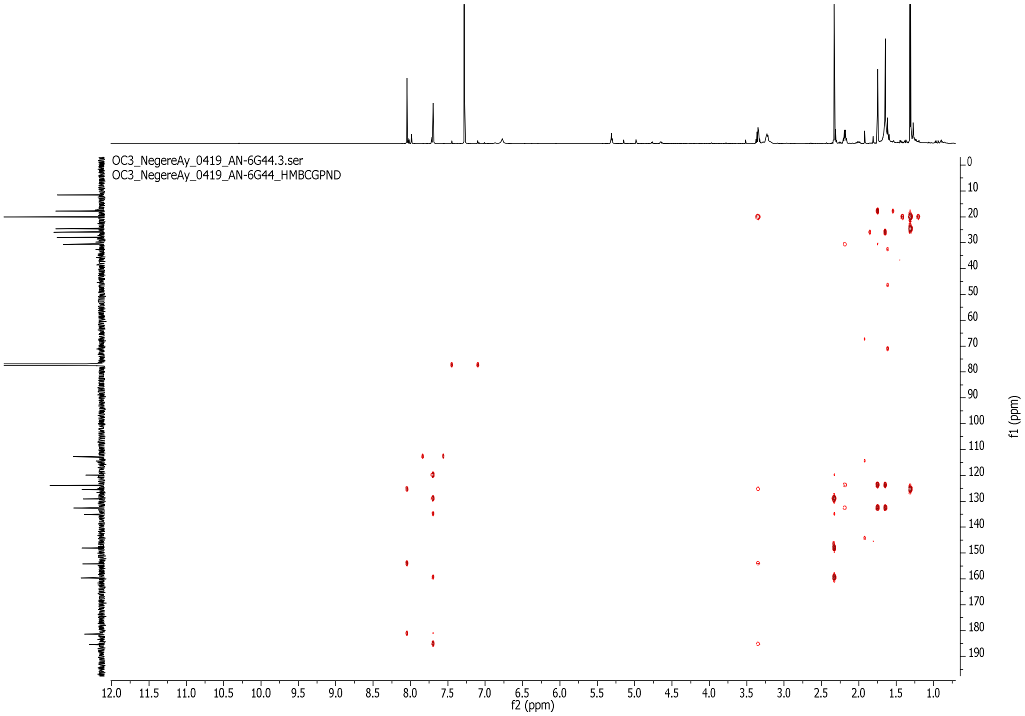

Figure S28. The HMBC spectrum of 6,12-dihydroxysapriparaquinone (**4**) observed at 500 and 125 MHz in $CDCl_3$ solution at 25 °C.

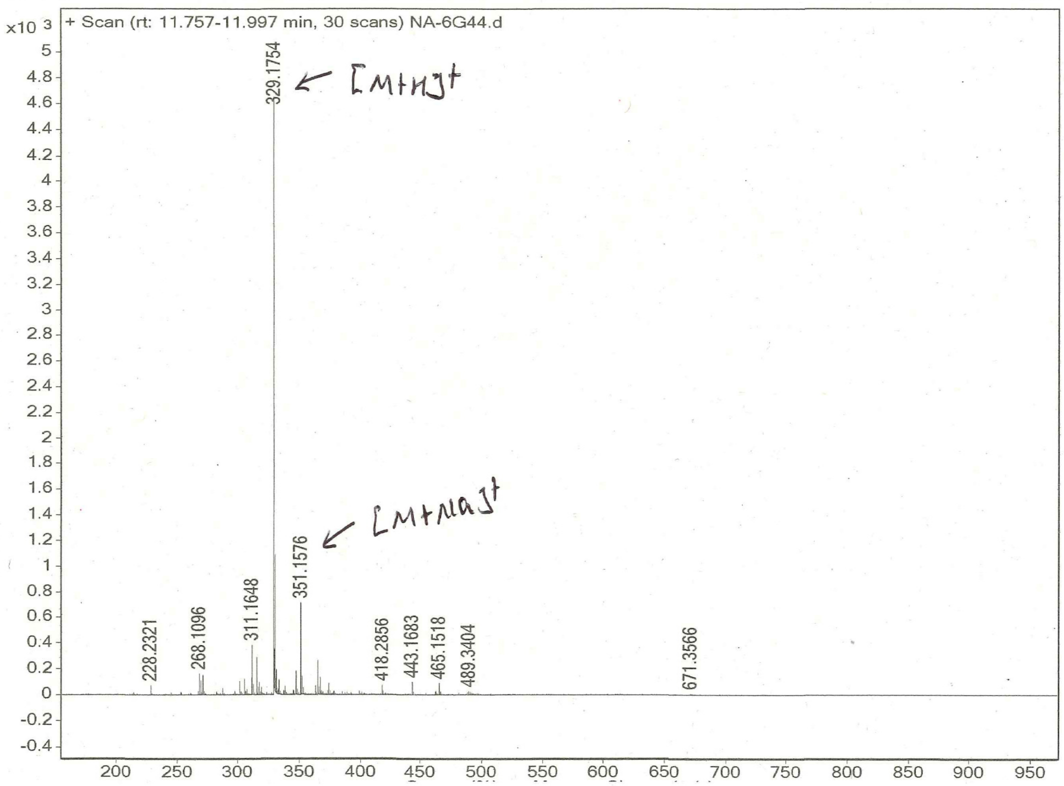

Figure S29: HRESIMS spectra of 6,12-dihydroxysapriparaquinone (**4**)
